# 3D-Printed, Ultrastretchable Polychloroprene Elastomers via Thiol-ene Photopolymerization

**DOI:** 10.1021/acsami.6c03614

**Published:** 2026-04-27

**Authors:** Levi M. J. Moore, Maren E. Summers, Ashley M. Robinson, Anesia D. Auguste, Reagan Elia, Jared A. Gibson, Jacob C. Marcischak, Johnnay A. Martin, Kamran B. Ghiassi

**Affiliations:** † Aerospace Systems Directorate, 97046 Air Force Research Laboratory, Edwards AFB, California 93524, United States; ‡ Amentum Holdings, Inc., Air Force Research Laboratory, Edwards AFB, California 93524, United States; § Materials and Manufacturing Directorate, 97042 Air Force Research Laboratory, Wright-Patterson AFB, Dayton, Ohio 45433, United States; ∥ AeroVironment, Inc., Air Force Research Laboratory, Wright-Patterson AFB, Dayton, Ohio 45433, United States; ⊥ United States Air Force Academy, Colorado Springs, Colorado 80840, United States

**Keywords:** elastomers, polychloroprene, thiol-ene, stretchability, 3D printing, vat photopolymerization

## Abstract

Polychloroprene is a foundational high-performance synthetic elastomer known for its exceptional chemical stability, resistance, and mechanical properties, making it useful in many important applications ranging from aerospace seals to medical devices. Despite its widespread use, polychloroprene is almost exclusively processed by using thermal cure agents. Herein, we report the first-ever successful ultraviolet (UV) curing and three-dimensional (3D) printing of polychloroprene networks. Leveraging thiol-ene click chemistry, solid polychloroprene was dissolved in a solvent and UV-cured with varied concentrations and architectures of thiol cross-linkers. Upon solvent evaporation, the resulting cross-linked polychloroprene networks exhibit ultrahigh extensibility, with strain at break values approaching 2000%. Significantly, their thermal properties show only marginal differences from those of the uncured material, confirming the preservation of the intrinsic polychloroprene characteristics. We demonstrate the potential of this new material platform using photorheology experiments, as well as successful 3D printing of complex objects using a commercial digital light processing (DLP) system. The printed articles exhibit mechanical properties fully comparable to conventionally processed, unfilled polychloroprene rubber, achieving an ultimate tensile stress of 2.4 MPa and a strain at break of 1200%. This work overcomes a significant processing barrier, offering an avenue to additively manufacture high-performance polychloroprene structures with exceptional mechanical resilience.

## Graphical abstract

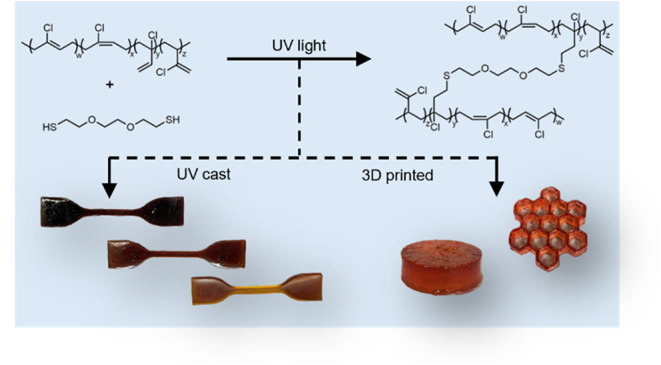

## 1 Introduction

As additive manufacturing technology has progressed and matured, there is an increased need for high-performance materials, especially low glass transition (*T*_g_) elastomers, for commercial applications.^1–5^ Although the flexible nature of elastomers presents a challenge to three-dimensional (3D) printing,^6,7^ certain classes of materials have been successfully processed using established additive manufacturing techniques. For instance, thermoplastic polyurethane (TPU) has been printed via fused filament fabrication (FFF),^8,9^ while rubber-like polyurethane acrylates have been fabricated using vat photopolymerization (VP) techniques such as stereolithography (SLA)^10,11^ or digital light processing (DLP).^12–14^ DLP, in particular, has the advantage of higher resolution, better adhesion between layers, and faster print speed^15^ compared to filament techniques^16^ due to entire layers being cured at the same time using a projected image.^17^ In the VP realm, however, it is difficult to print elastomers due to the high molecular weights required to achieve suitable mechanical properties for use in high-performance applications. Many approaches have been reported including scaffolding from latexes^18,19^ and formulating with reactive diluents^20^ with generally good results. However, the mechanical properties of compounded rubber remain difficult to achieve.^21,22^

A formulation solution to this elastomer problem would be advantageous, since unmodified commercial elastomer feedstocks are commodities and inexpensive compared to products explicitly modified to contain (meth)­acrylate groups that are designed to be UV-curable and/or 3D-printable.^23^ The addition of (meth)­acrylate groups also leads to environmental instability, where extended storage can lead to autopolymerization and batch-to-batch variation.^24^ Polychloroprene was chosen for this study due to its commercial availability, low glass transition temperature, and intrinsic unsaturated moieties capable of rapid cross-linking with UV-promoted thiol-ene chemistry.^25^ Polychloroprene is typically thermally cross-linked using a metal oxide, such as ZnO or MgO, in conjunction with an accelerator like ethylene thiourea.^26^ A number of reports utilized the thiol-ene reaction to cure, functionalize, or 3D print polybutadiene,^27–29^ but few reports use the thiol-ene reaction for polychloroprene rubber for either functionalization^30^ or thermal cross-linking.^31^

In this report, we extend our previous framework using a multifunctional thiol as the cross-linking agent in DLP 3D printing^28^ to polychloroprene rubber. This is the first report of using UV light to cross-link polychloroprene by casting or additive manufacturing. Using solvent-assisted thiol-ene click chemistry, we synthesized polychloroprene elastomer networks that demonstrate exceptional toughness and stretchability after UV casting with strain at break values approaching 2000%. The resin formulations could also be 3D printed using a commercial DLP printer, resulting in properties comparable to those of conventionally processed polychloroprene. This research enables the 3D printing of high-performance polychloroprene structures with outstanding toughness and extensibility, overcoming a substantial processing barrier.

## 2 Experimental Section

### 2.1 Materials

Polychloroprene (2-chloro-1,3-butadiene polymer, Baypren 211 M39) was obtained from ARLANXEO. Xylenes, trimethylolpropane tris­(3-mercaptopropionate) (TMPMP), pentaerythritol tetrakis­(3-mercaptopropionate) (PETMP), and Oil Red O were purchased from Sigma-Aldrich. Diphenyl­(2,4,6-trimethylbenzoyl)­phosphine oxide (TPO), 3,6-dioxa-1,8-octanedithiol (ODT), and decanethiol (DT) were purchased from TCI. All materials were used as received. A FlackTek SpeedMixer DAC 600 FVZ instrument was used for all mixing. Cast articles were cured in a Formlabs Form Cure at 405 nm for 10 min.

### 2.2 Preparation of Photoresins

A 16% by mass solution of polychloroprene in xylenes (a 5:1 ratio of xylenes/polychloroprene by mass), a thiol (DT/ODT/TMPMP/PETMP), and a photoinitiator (TPO) were added to a polypropylene mixing cup and mixed in a dual-axis centrifuge for 2 min at 2300 rpm. To prepare the resin for vat photopolymerization, the previously mentioned procedure was followed, but the photoabsorber solution was added to the mixing cup before mixing. This photoabsorber solution was a 0.025 M solution of Oil Red O in xylenes. Along with the four different thiols studied, three different thiol loadings were investigated for each individual thiol. Photoresins were made to have either 30, 60, or 90 thiols per polymer chain. For example, to prepare an ODT photoresin for 3D printing with 90 SH groups per polymer chain, 40 g of polychloroprene in xylenes, 0.304 g of ODT, 0.070 g of TPO, and 273 μL of 0.025 M Oil Red O solution were measured into a mixing cup and mixed in a dual-axis centrifuge for 2 min at 2300 rpm.

### 2.3 Tensile Sample Preparation and Measurement

A mold for tensile samples was printed based on ASTM D412 Die C dimensions (dumbbell-shaped specimens with a 25 mm gauge length and 2.79 mm width in the gauge length area). The samples underwent isotropic shrinkage after casting, resulting in dimensions that no longer followed that of ASTM D412. The thickness of the mold was increased by 5 mm to ensure the samples were still thick enough for mechanical testing after shrinking. The mold was printed in an UltiMaker S5 instrument with PETG as the filament. Tensile samples were prepared by pouring undyed photoresin into the mold and curing for 10 min at 405 nm and 9.1 W in a Formlabs FormCure at room temperature. Dogbones were demolded after curing, and solvent was allowed to evaporate in a vent hood for 7 days. Solvent removal was completed by placing the dogbones in a 60 °C oven overnight.

Tensile properties were determined according to ASTM D412-16, Method A, using an Instron-68TM-5 load frame equipped with a 100 N load cell. At least six specimens per sample variable were tested at a strain rate of 500 mm/min at 23 °C and 28% relative humidity. Elongation was measured using a digital video extensometer (Instron AVE2) by tracking the displacement of two white benchmarks placed 20 mm apart on each specimen.

### 2.4 Thermal and Physical Property Measurements

Viscosity values were recorded at 25 °C on a Brookfield DVNext Cone/Plate rheometer. Nuclear magnetic resonance (NMR) experiments were performed on a Bruker AVANCE III HD 400 MHz spectrometer using CDCl_3_ or C_6_D_6_ as the solvent. Fourier transform infrared (FTIR) data were acquired on a Nicolet iS50 spectrometer equipped with an attenuated total reflectance (ATR) accessory with an acquisition window ranging from 600 to 4000 cm^–1^ at 4 cm^–1^ resolution. Uncured samples were measured by dissolving the formulation in dichloromethane and drop casting onto the ATR accessory, resulting in some residual dichloromethane present in the spectra (signals at 750 and 1260 cm^–1^). Gel fraction data were determined by soaking each preweighed sample in 5 mL of chloroform for 3 days, replacing the liquid with fresh chloroform every 24 h to ensure complete removal of the soluble fraction. The surface of the swelled sample was patted dry with a Kimwipe and weighed to determine the swelling ratio. The insoluble fraction was then dried under a 20 mTorr vacuum for 3 days before being reweighed. The gel fraction was calculated as the mass of the dried insoluble fraction divided by the initial mass of the sample. Differential scanning calorimetry (DSC) data were generated on a TA Instruments DSC2500. Experiments used a heat/cool/heat cycle with the temperature limits of −75 to 250 °C, a heat rate of 10 °C/min, a cool rate of 5 °C/min, and a nitrogen sample purge rate of 50 mL/min. Thermogravimetric analysis (TGA) data were generated on a TA Instruments TGA5500. Experiments were run in nitrogen from 25 to 600 °C with a heat rate of 10 °C/min. Shore A values were measured on an insize testing stand with a Shore A durometer.

### 2.5 Microstructure Analysis from NMR Data

Samples of polychloroprene, polychloroprene cured with TPO, and the soluble fraction of polychloroprene cured with TPO and decanethiol were analyzed by ^1^H NMR spectroscopy in C_6_D_6_ following the procedure outlined by Makhiyanov.^32^ The ratio of aliphatic to olefinic protons was calculated for each sample by dividing the integral intensity for the range of 3.0–1.8 ppm from the integral intensity for the range of 6.0–4.8 ppm. The mole fractions of *cis*-, *trans*-, and *vinyl*-groups were calculated for each sample by comparing the integral intensities for the peaks at 5.5 ppm (*cis*-), 5.25 ppm (*trans*-), and 5.75 and 5.05 ppm (*vinyl*-).

### 2.6 Photorheology Measurements

Photorheology experiments were conducted on a TA Instruments Discovery Series HR-3 equipped with a Smart Swap UV assembly with a 20 mm aluminum upper plate, a 20 mm quartz bottom plate, and an Omnicure S2000 high-pressure mercury light source (320–500 nm filter). UV intensity was calibrated with a Silverline radiometer and a 20 mm sensor attachment for the quartz parallel plate. All experiments had a 100 μm gap size, 0.1% strain, and 5 Hz frequency. UV radiation was applied for 60 s after a 30 s delay with an intensity of 1 mW/cm^2^. Data were collected in triplicate from separate formulation batches to ensure reproducibility. Plateau storage moduli values were determined from the final 20 s of each data set.

### 2.7 Vat Photopolymerization

All 3D printing experiments were performed on a FlashForge Hunter S Dental 3D Printer (FlashForge USA, City of Industry, CA). Standard Tessellation Language files were processed in FlashDLPrint. All files were sliced to a layer thickness of 100 μm. All layers received 405 nm light set to 100% light intensity. When measured with a Silverline radiometer, 100% light intensity corresponded to approximately 1 mW/cm^2^. The initial three layers of each print received 100 s of exposure to ensure proper attachment of the print to the build plate. The remaining layers were exposed for 20 s. Prints were performed with 40–100 g of photoresin, depending on the size of the print. For taller prints, the vat was refilled with more photoresin when the resin level was running low by pausing the print between layers. Prints were removed from the build plate with a metal scraper. Any excess resin was cleaned off with xylenes and a Kimwipe. To quantify article shrinkage, cubes were printed in various sizes. Initial measurements were taken in the *x*, *y*, and *z* directions immediately after removing the print from the build plate. Cubes were left in a fume hood to dry for 2 weeks before being transferred to a 60 °C oven overnight. Final measurements were taken immediately after the cubes were taken out of the oven.

## 3 Results and Discussion

### 3.1 Tuning the Resin Viscosity

A viscosity of ca. 5 Pa·s or lower is optimal for vat photopolymerization.^2^ This requirement presents a challenge for polychloroprene. Unlike polybutadiene, which is available in low molecular weight liquid oligomeric form, polychloroprene is only available as a high molecular weight solid (minimum reported *M*_n_ of 180 kg/mol), making direct VP unfeasible. This necessitated the dissolution of the polymer in a solvent to make it processable via vat photopolymerization. In this study, xylenes were chosen as the solvent for this process due to their ability to easily dissolve polychloroprene and their relatively high boiling point, which was desired to reduce the amount of solvent lost to evaporation during the printing process. A 5:1 mass ratio of xylenes to polychloroprene provided a good balance of solid content and processable viscosity of ca. 0.2 Pa·s (Table S2).

### 3.2 Elucidating the Cross-linking Mechanism

Thiol-ene click chemistry was used to cross-link the resins upon exposure to UV light. To assess where the thiol adds on the polymer backbone (i.e., the *trans*-, *cis*-, or two different *vinyl*-moieties^26^), decanethiol (DT) was added to the polychloroprene/xylenes solution with a photoinitiator and irradiated with 405 nm light. In our prior work, NMR analysis of the reaction between a monothiol and polybutadiene showed preferential addition to the 1,2-vinyl moieties.^28^ A previous report investigated the addition of thioacetic acid to polychloroprene using a thermal thiol-ene reaction although the results were inconclusive as to the exact location of thiol addition.^30^ Surprisingly, in the polychloroprene case reported here, the mixture solidified after exposure to UV light. After the solid was soaked in chloroform for 3 days, 10–20% of each sample was able to dissolve (Table S4). NMR analysis was performed on the soluble fraction to elucidate the location of the monothiol addition (Table S3). The ratio of aliphatic to olefinic protons increased from 3.6 to 3.8 after functionalization due to the grafting of decanethiol. However, there was no observable change in the ratio of *cis*-, *trans*-, and *vinyl*-protons, signifying no preference for the location of thiol addition. The presence of an insoluble fraction suggests that another cross-linking mechanism may also be at play, such as direct radical cross-linking of backbone alkenes, though the exact mechanism is unclear at this time. To examine this, a similar experiment was performed where only a photoinitiator was added to the polymer solution. This mixture also gelled slightly although it remained soluble in chloroform. The ratio of aliphatic to olefinic protons increased very slightly, from 3.6 to 3.7, supporting our hypothesis of direct radical cross-linking of polychloroprene in the presence of a radical photoinitiator.

### 3.3 Synthesis and Design of Tunable Polychloroprene Networks

Figure 1 shows the selection of materials used in this study to interrogate the effect of cross-linker type and loading on the mechanical properties of UV-cured polychloroprene. Several multifunctional thiols, 3,6-dioxa-1,8-octanedithiol (ODT), trimethylolpropane tris­(3-mercaptopropionate) (TMPMP), and pentaerythritol tetrakis­(3-mercaptopropionate) (PETMP) were chosen to examine the effect of thiol functionality on mechanical properties. Three loadings were chosen at equimolar amounts of thiol functionality, referred to herein as 30, 60, and 90 SH/chain. These loadings correspond to an average molecular weight between thiol cross-links of approximately 6000, 3000, and 2000 g/mol, respectively. Maintaining these ratios as a constant across all formulations allows for direct comparison of the mechanical response to the change in network topology.

**Figure 1:**
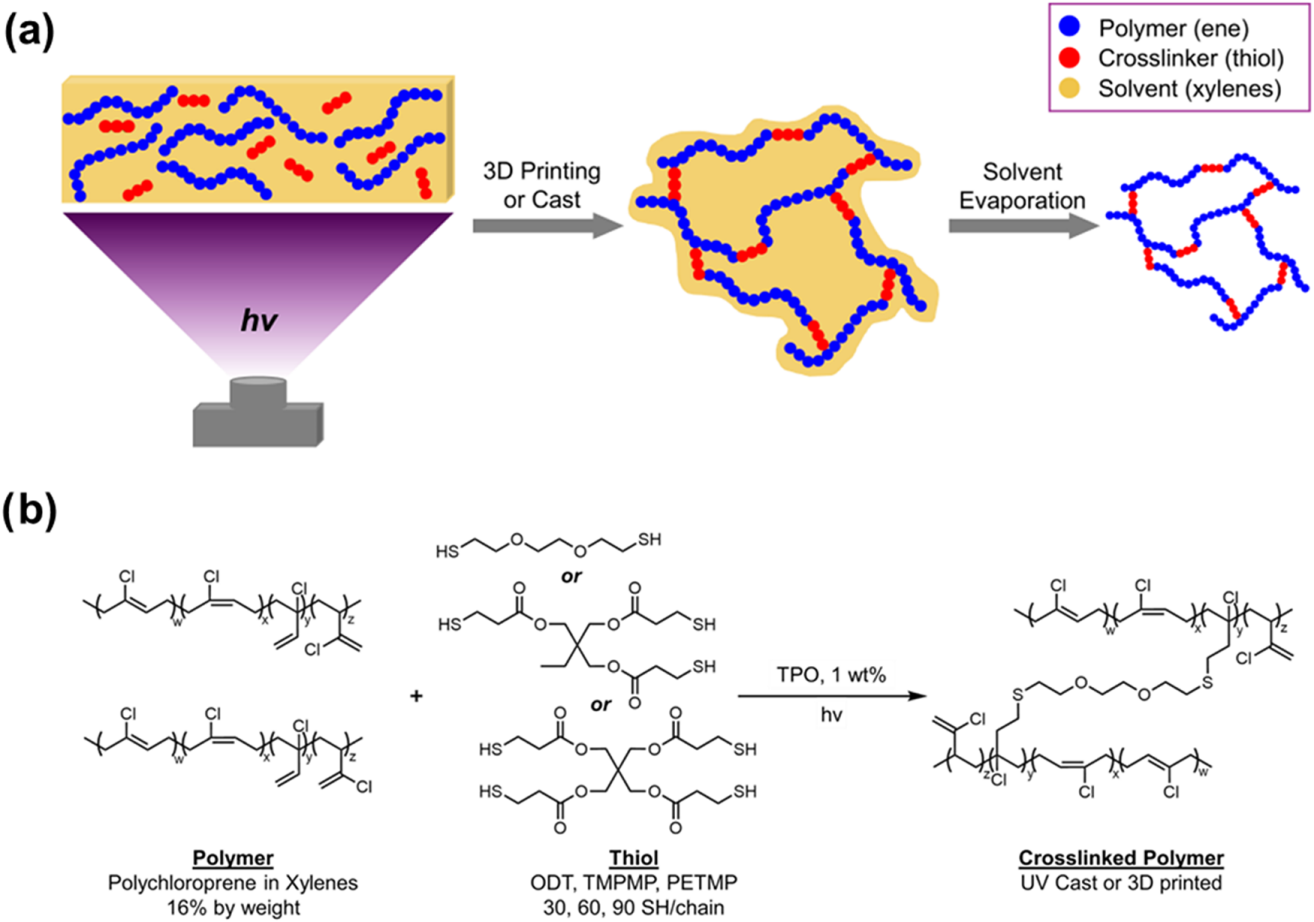
(a) Cartoon depicting the solvent-assisted photopolymerization method; (b) materials used in this study and a notional cross-linking scheme employing a dithiol.

Specimens for material characterization and tensile testing were cast by using the ASTM D412C geometry. A mold was 3D printed using a conventional FFF printer and used to UV cast the formulations from solution. Resins were poured into the mold and cured for 10 min at 405 nm in a Formlabs FormCure. The articles were demolded and left to dry at ambient conditions for 7 days, followed by an oven at 60 °C overnight. Interestingly, a darkening effect was observed as the solvent evaporated, with lower loadings exhibiting a darker appearance (Figure S19). The underlying cause of this phenomenon is still under investigation. The samples were then subjected to material characterization, as summarized in Table 1.

**Table 1:** Properties of UV-Cross-linked Polychloroprene with Different Thiols and Thiol Loadings

thiol	thiol loading [SH/chain]	gel fraction [%]	*T*_g_ [°C]	*T*_d_ (5%), N_2_ [°C]
starting PC			–38.7 ± 0.3	267
ODT	30	91.2 ± 0.7	–37.9 ± 1.0	256
	60	93.8 ± 0.8	–38.4 ± 1.1	247
	90	94.1 ± 0.2	–38.4 ± 1.2	236
TMPMP	30	95.0 ± 0.7	–34.8 ± 1.7	261
	60	94.8 ± 0.9	–35.2 ± 1.8	261
	90	96.7 ± 0.7	–34.3 ± 1.2	254
PETMP	30	94.7 ± 0.5	–35.4 ± 1.1	250
	60	96.8 ± 0.3	–34.8 ± 0.7	254
	90	97.0 ± 0.5	–35.1 ± 0.2	251

### 3.4 Characterization of UV-Cast Networks

#### 3.4.1 Cross-linking Conversion

FTIR spectroscopy of the resins before and after curing was measured to determine the conversion of the thiol-ene reaction. All samples contain signals at 830 cm^–1^ (CH_2_ rocking), 1110 cm^–1^ (C–C stretch), 1430 cm^–1^ (CH_2_ deformation), 1660 cm^–1^ (C=C stretch), and 2800–3000 cm^–1^ (C–H stretch) as shown in Figures S10–S17. Unfortunately, FTIR spectroscopy was found to be unsuitable to measure the conversion of the thiols during cure due to the low intensity of the S–H stretch around 2600 cm^–1^ in the unreacted resins. FTIR analysis of the alkene reactivity was similarly inconclusive with no obvious change in the intensities of known signals after curing. Instead, to estimate conversion, the gel fraction was measured for each cured resin formulation after soaking a sample in chloroform three subsequent times for 24 h each time. The results shown in Table 1 are an average of three specimens. Gel fraction increases slightly as thiol loading increases and as thiol functionality increases, which is expected from a more cross-linked network. However, all formulations had 91% or greater gel content, consistent with other reported thiol-ene resins for VP, suggesting sufficient conversion to form an insoluble network.^33^

#### 3.4.2 Thermal Properties

Differential scanning calorimetry (DSC) was used to measure the thermal transitions of the various polymer networks. The glass transition temperature does not substantially change from the pristine uncross-linked material, seen in Figure 2A and Table 1. There is a slight increase in the *T*_g_ for networks containing ester-based cross-linkers (−35 °C instead of −38 °C) attributed to hydrogen bonding from the incorporated ester moieties. Decomposition temperatures observed with TGA (Figure 2B) are lowered slightly upon cross-linking, most likely due to the increased hydrocarbon fraction that incorporates the weaker C–S bond. Holistically, cross-linking the polychloroprene material using UV-promoted thiol-ene chemistry does not seem to significantly change the thermal properties of polychloroprene. We attribute this to the comparatively high molecular weight between cross-links compared to other cross-linked materials. Most of the polymer structure remains unchanged after cross-linking, resulting in similar thermal properties.

**Figure 2:**
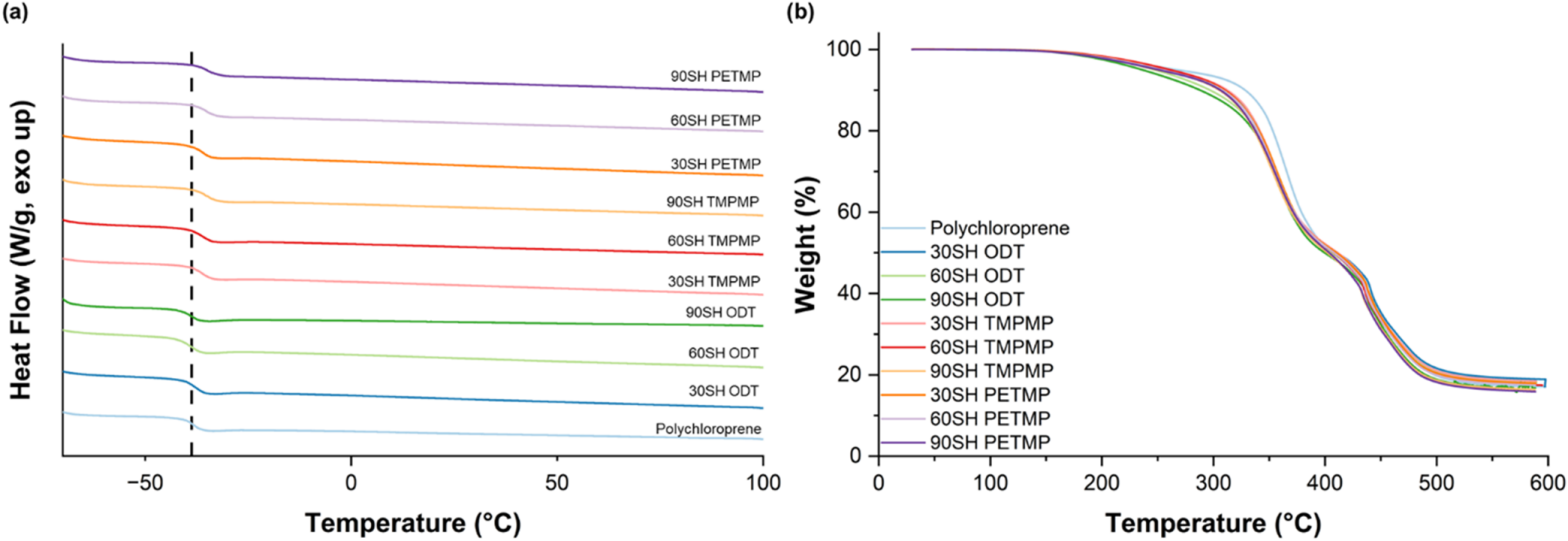
(a) DSC thermograms and (b) TGA plot in N_2_ of all cured formulations and feedstock polychloroprene.

#### 3.4.3 Mechanical Properties

The uniaxial tensile properties were determined following the procedure outlined in ASTM D412-16 Method A using an Instron-68TM-5 load frame with a 100 N load cell and at a rate of elongation of 500 mm/min. While samples of polychloroprene with a photoinitiator gelled slightly as discussed previously, they were not robust enough for tensile testing. Formulations containing decanethiol showed excellent mechanical properties, with over 2000% strain at break and over 3 MPa ultimate tensile stress (UTS), despite the presence of 10–20% soluble content (Table S5). Future work will investigate the mechanism and material properties of decanethiol-cured polychloroprene. The tensile tests of conventional thiol cross-linkers, summarized in Figure 3 and Table S5, also demonstrate superior extensibility, with some formulations showing ca. 2000% strain at break with up to 8 MPa UTS. While the tensile strength is within the typical range (3.4–20 MPa) for unfilled, conventionally cured polychloroprene rubbers, the observed strain at break behavior is notably higher than their characteristic range of 100–800%.^34–36^ Remarkably, several of these formulations, tested at a rate of elongation of 500 mm/min, outperform the extensibility of many state-of-the-art elastomers and tough hydrogels even when the latter are tested at significantly lower rates of elongation.^2,37–41^ This suggests a highly efficient energy dissipation mechanism within the thiol-ene cross-linked architecture that defies the typical trade-off between the loading rate and ductility.

**Figure 3:**
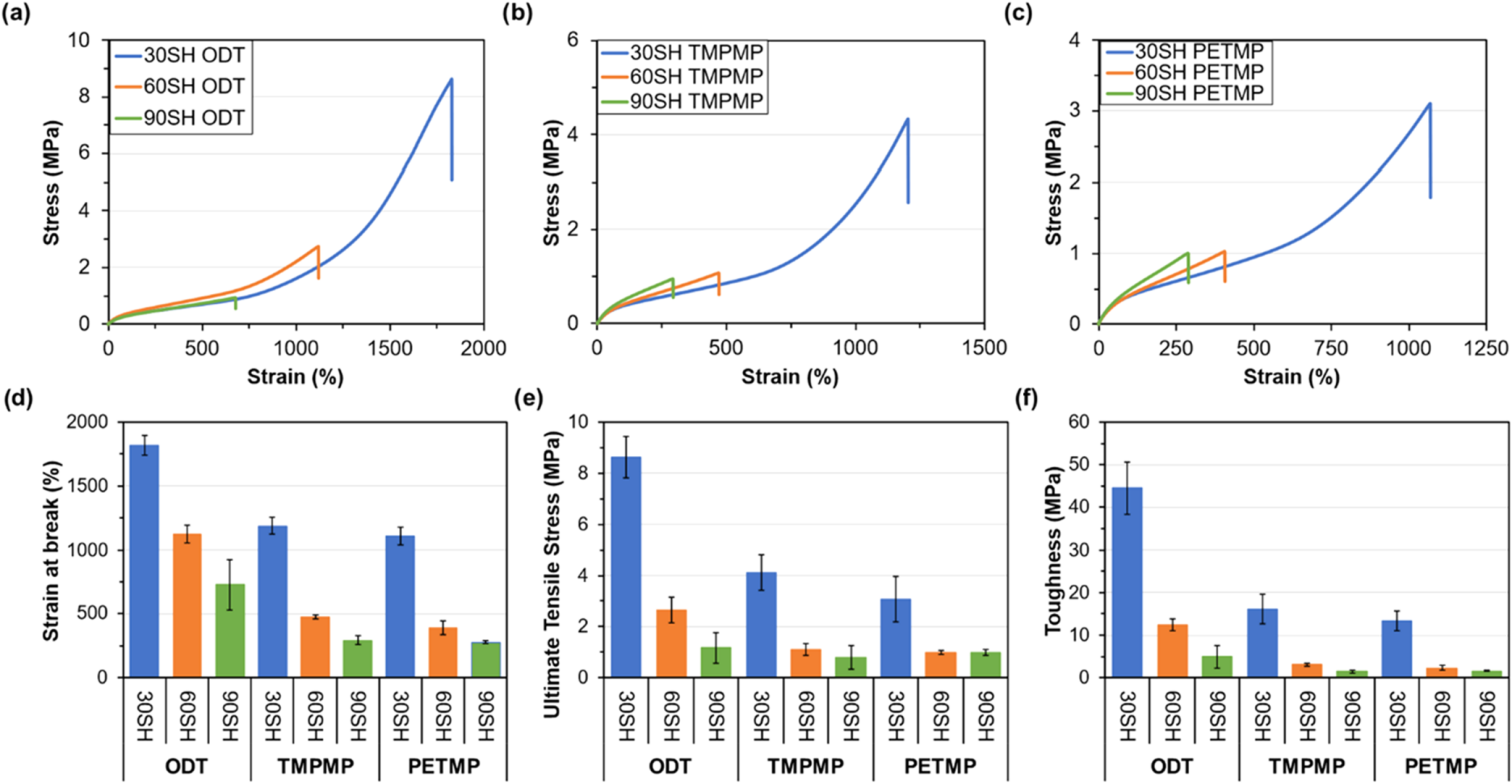
Top: stress and strain curves for the UV-cast polychloroprene elastomer samples, grouped by thiol type, show differences with respect to thiol loading. From left to right: (a) ODT, (b) TMPMP, and (c) PETMP. Note differences in scale in the *x*- and *y*-axes. Bottom: bar graphs of the mechanical properties of UV-cast polychloroprene elastomer samples. From left to right: (d) strain at break, (e) ultimate tensile stress, and (f) toughness.

A systematic investigation of cross-linker concentration revealed an inverse correlation with mechanical performance, such that when the cross-linker loading decreased, both the UTS and material toughness increased. For example, the 30 SH ODT formulation has a toughness of 44.6 ± 6.1 MPa, which is almost 900% greater than the 90 SH ODT formulation (5.0 ± 2.7 MPa) even though it contains a third of the cross-linker. As cross-linker loading decreases, the material exhibits greater elongation, facilitating strain-induced crystallization and subsequent strain hardening, reflected in the observed upturn of the stress–strain curve at high strains. This is due to the alignment of the polychloroprene chains as the mesh size (or cross-link distance) increases from lower cross-linker loading, ultimately leading to a higher tensile stress. The UTS and material toughness also tend to decrease as the functionality of the cross-linker is increased, from di- to tri- to tetrafunctional, as the thiol changes from ODT to TMPMP to PETMP. This suggests that the cross-linker structure and resulting network topology strongly influence the mechanical properties. For example, the 90 SH ODT formulation, incorporating a difunctional cross-linker, has a UTS of 1.18 ± 0.5 MPa and toughness of 5.0 ± 2.7 MPa, while the formulation incorporating the tetrafunctional cross-linker PETMP has 0.99 ± 0.1 MPa and toughness of 1.6 ± 0.1 MPa. The thiol concentration is the same between the two formulations; therefore, the resulting difference is strictly attributed to the presence of difunctional versus tetrafunctional junction points between polymer chains.

### 3.5 Photorheology

Photorheology experiments were conducted on each formulation to estimate cure kinetics and mechanical properties after 3D printing (Figure 4). The time it takes for the storage modulus to exceed the loss modulus after 1 mW/cm^2^ UV light was introduced, known as the crossover time, was measured to estimate the curing rate. The plateau shear storage modulus was also measured to indicate the structural rigidity of the network after 60 s of UV exposure. Results are listed in Table 2. A plateau modulus of at least 50 kPa is desired for successful 3D printing. The polychloroprene formulations had plateau storage moduli of 5–8 kPa, suggesting the possibility of structural defects in 3D-printed articles. Photorheology of the polymer with photoinitiator but no thiol cross-linker demonstrated a slight increase in storage modulus (7–20 Pa), but no crossover time was observed (Figure S32). However, when decanethiol was used in place of the thiol cross-linker, the sample completely solidified with a crossover time of 20–27 s and a plateau storage modulus of 1 kPa (Figure S33). For all four thiol cross-linkers, increasing the thiol loading from 30 to 60 to 90 SH/chain did not influence the resulting crossover time or plateau storage modulus (Figures S34–S36). This indicates that changing the thiol cross-linker concentration has no appreciable effect on cure rate or the structural rigidity of the final network. Increasing the thiol cross-linker functionality from 2 to 3 resulted in decreased crossover time (12–9 s) and increased plateau storage modulus (5–8 kPa). Therefore, when the number of functional groups on the thiol cross-linker is increased, the cure rate and structural rigidity of the final network will also increase.

**Figure 4:**
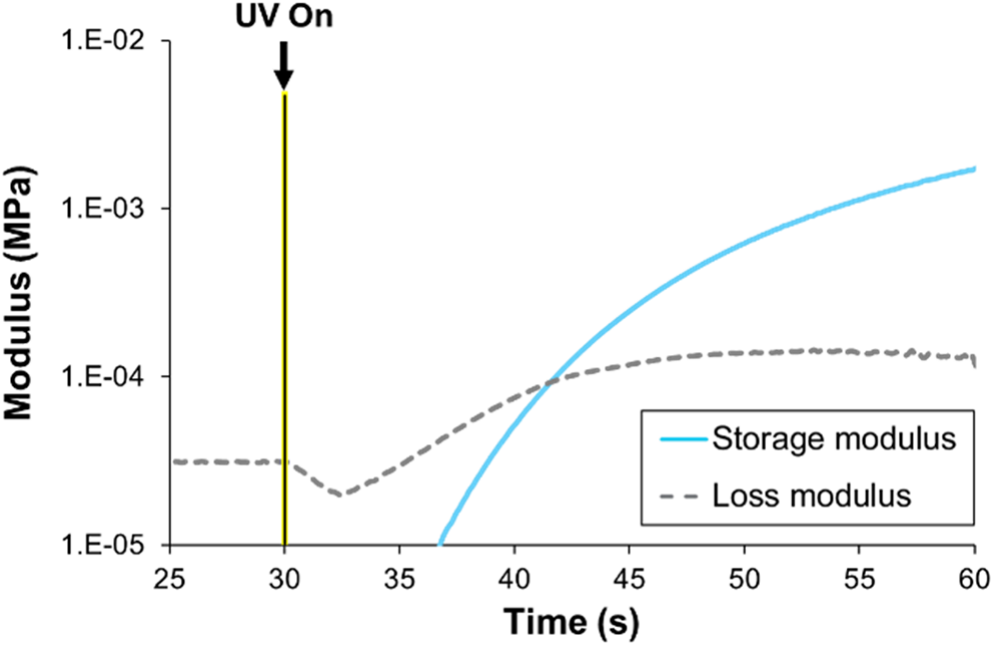
Representative photorheology experiment of PC with 90 SH ODT cross-linker.

**Table 2:** Photorheology of UV-Cross-linked Polychloroprene with Different Thiols and Thiol Loadings^a^

thiol	thiol loading [SH/chain]	crossover time [s]	plateau storage modulus [kPa]
none			0.02 ± 0.01
DT	30	27 ± 4	0.8 ± 0.3
	60	19 ± 1	1.1 ± 0.7
	90	20 ± 1	1.6 ± 0.3
ODT	30	13 ± 1	4.6 ± 1.4
	60	12 ± 0.3	4.8 ± 0.3
	90	12 ± 1	4.6 ± 1.2
TMPMP	30	9 ± 1	8.1 ± 0.6
	60	9 ± 1	7.5 ± 1.8
	90	9 ± 0.4	7.8 ± 0.5
PETMP	30	9 ± 1	8.0 ± 1.8
	60	9 ± 1	8.1 ± 0.6
	90	10 ± 1	5.6 ± 1.1

a Plus or minus values represent 1 standard deviation from a minimum of three samples.

### 3.6 3D Printing

All UV-cast formulations were printed using a FlashForge Hunter S Dental 3D Printer with a layer thickness of 100 μm and a layer exposure time of 20 s. Similar to our previous report using polybutadiene,^28^ the formulations that were only polymer solution, cross-linker, and photoinitiator experienced excessive light bleed and overcure into the vat, leading to poor dimensional resolution. Oil Red O was added as a light absorber at 0.04 wt % (optimized) with respect to polychloroprene. This was highly effective in reducing the amount of overcure observed, resulting in printed parts with good dimensionality (Figure S37). Decanethiol formulations could not be successfully printed due to the low structural rigidity of the cured networks, which resulted in significant defects during printing and upon removal from the build plate, as shown in Figure S38.

After an article was removed from the build plate, it was placed in a hood to allow the xylenes to evaporate, resulting in shrinkage. To quantify shrinkage after printing, 1 and 2 cm cubes were printed to allow for facile tracking of dimensional changes along all three planes via camera (Figure S39, Table S6). The 90 SH formulation with ODT as the cross-linker was used for this test. The final density of the 1 and 2 cm cubes was 1.17 ± 0.06 and 1.19 ± 0.06 g/cm^3^, respectively, approaching the calculated density of cured bulk material of 1.23 g/cm^3^. For the 1 and 2 cm cubes, the prints’ side lengths both shrank by roughly 46% and volumes by 85%. Most of the volume was lost within 24 h and can be seen in a time-lapse video in the Supporting Information. These values were consistent with the amount of solvent present in the photoresin since the polychloroprene in xylenes solution was 88% solvent by volume. As shrinkage occurred, the materials became tougher and darker in color. The volume reduction is quite significant for these photoresin formulations and must be considered when designing a final printed article. Reduced shrinkage could be achieved by decreasing the amount of solvent; however, this would impact the viscosity of the polychloroprene solution and its ability to print successfully. If the resin is too viscous, it will have trouble recoating the vat between print layers, which could lead to failed prints. This could be mitigated by also increasing the temperature of the resin during printing to lower viscosity. Alternatively, a lower molecular weight polychloroprene could be synthesized to lower resin viscosity without resulting in significant shrinkage, but the mechanical properties of the resulting networks would likely suffer due to the reduced number of chain entanglements.

#### 3.6.1 Mechanical Properties of the 3D-Printed Articles

Mechanical properties of the printed formulations were measured by using uniaxial tensile tests of articles printed in the ASTM D412C geometry (Figure 5, Table S7). In all cases, the properties of the printed samples were reduced compared to their cast counterparts, exhibiting a significant trade-off in performance likely due to the additive manufacturing process. Specifically, the strain at break was reduced from 1800% (cast) to 1200% (printed), and the UTS decreased from over 8 MPa (cast) to just over 2 MPa (printed) (Figure 5A–C). This reduction in value and the increase in error (Figure 5D–F) can be primarily attributed to an increase in print defects (e.g., voids, layer nonadhesion) compared to the homogeneously cast samples, which act as stress concentration sites. Despite the reduction from cast samples, the measured UTS and stress–strain behavior of the 3D-printed articles remain comparable to unfilled, conventionally cured polychloroprene rubbers.^34,35^ The established trends observed in the cast samples are maintained in the 3D-printed versions. Both the UTS and the material toughness tend to increase as the cross-linker loading decreases. For example, 90 SH ODT has a toughness of 2.98 ± 0.30 MPa but increases over 460% to 13.9 ± 7.9 MPa in the lower cross-linker loading 30 SH ODT formulation. Conversely, the UTS and material toughness generally decrease as the functionality of the cross-linker is increased (from di- to tri- to tetrafunctional). For instance, the difunctional 90 SH ODT formulation exhibited a UTS of 0.97 ± 0.05 MPa and toughness of 2.98 ± 0.30 MPa, while the tetrafunctional PETMP counterpart showed a lower UTS of 0.91 ± 0.09 MPa and significantly reduced toughness of 1.30 ± 0.32 MPa.

**Figure 5:**
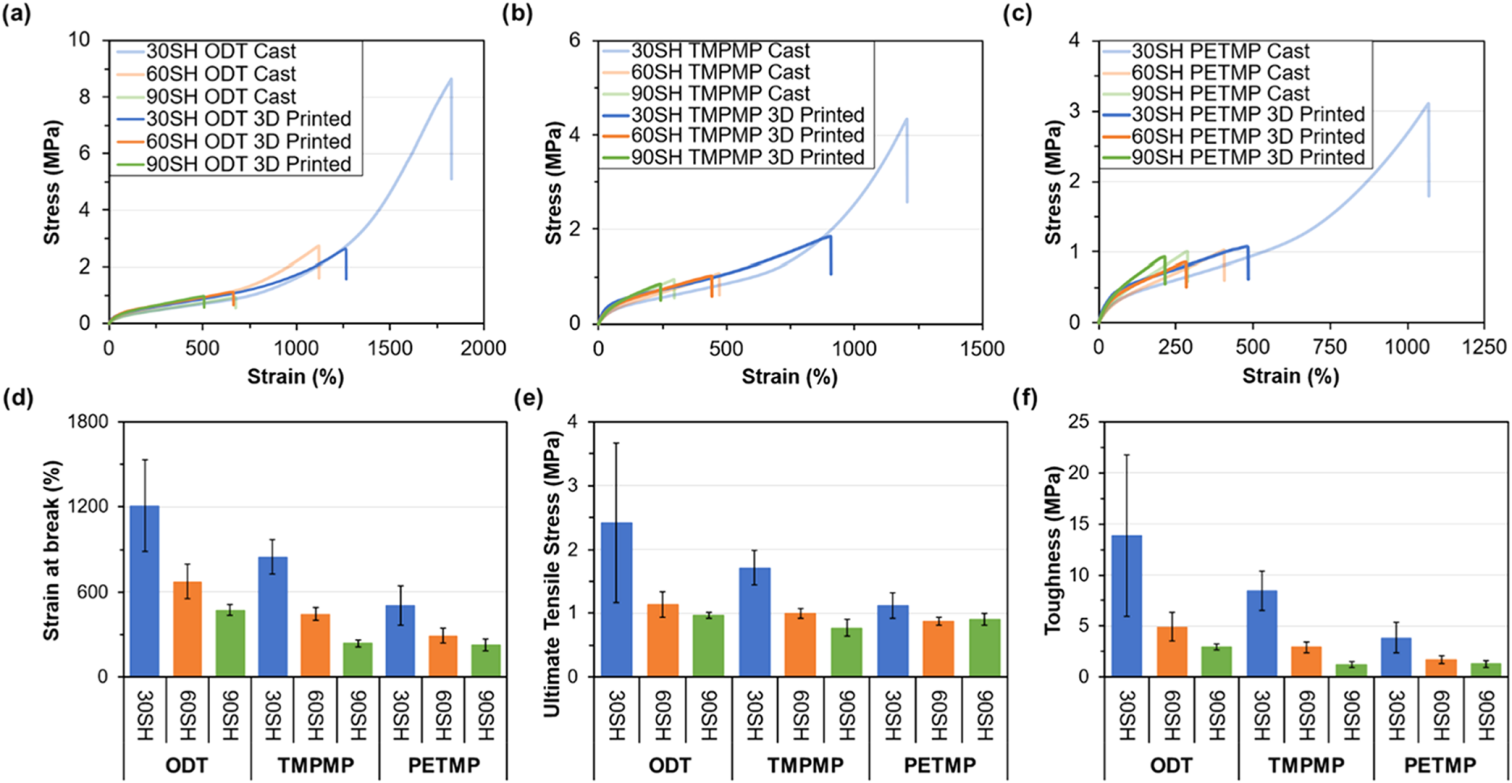
Top: stress and strain curves for the UV-cast (light) and 3D-printed (dark) polychloroprene elastomer samples are grouped by thiol type and show differences with respect to thiol loading. From left to right: (a) ODT, (b) TMPMP, and (c) PETMP; 30 SH, 60 SH, and 90 SH formulations are blue, orange, and green, respectively. Note differences in scale in the *x*- and *y*-axes. Bottom: bar graphs of the mechanical properties of the 3D-printed polychloroprene elastomer samples. From left to right: (d) strain at break, (e) ultimate tensile stress, and (f) toughness.

#### 3.6.2 Printing Complex Shapes

The 90 SH formulation with ODT as the cross-linker was selected as the primary resin based on its mechanical properties and was used to demonstrate more complex printed articles. Despite the large amount of solvent used in the resin formulation, the resulting printed geometries were robust enough to withstand the mechanical forces of the printing process, being raised and lowered out of the vat, as well as postprint processing. Simple cylinders were utilized as test prints, leading to more complex geometries including a hexagonal lattice and a twisted prism, shown in Figure 6. The prints exhibited isotropic shrinkage similar to previous observations, corresponding to an approximately 85% reduction in volume as the solvent evaporated.

**Figure 6:**
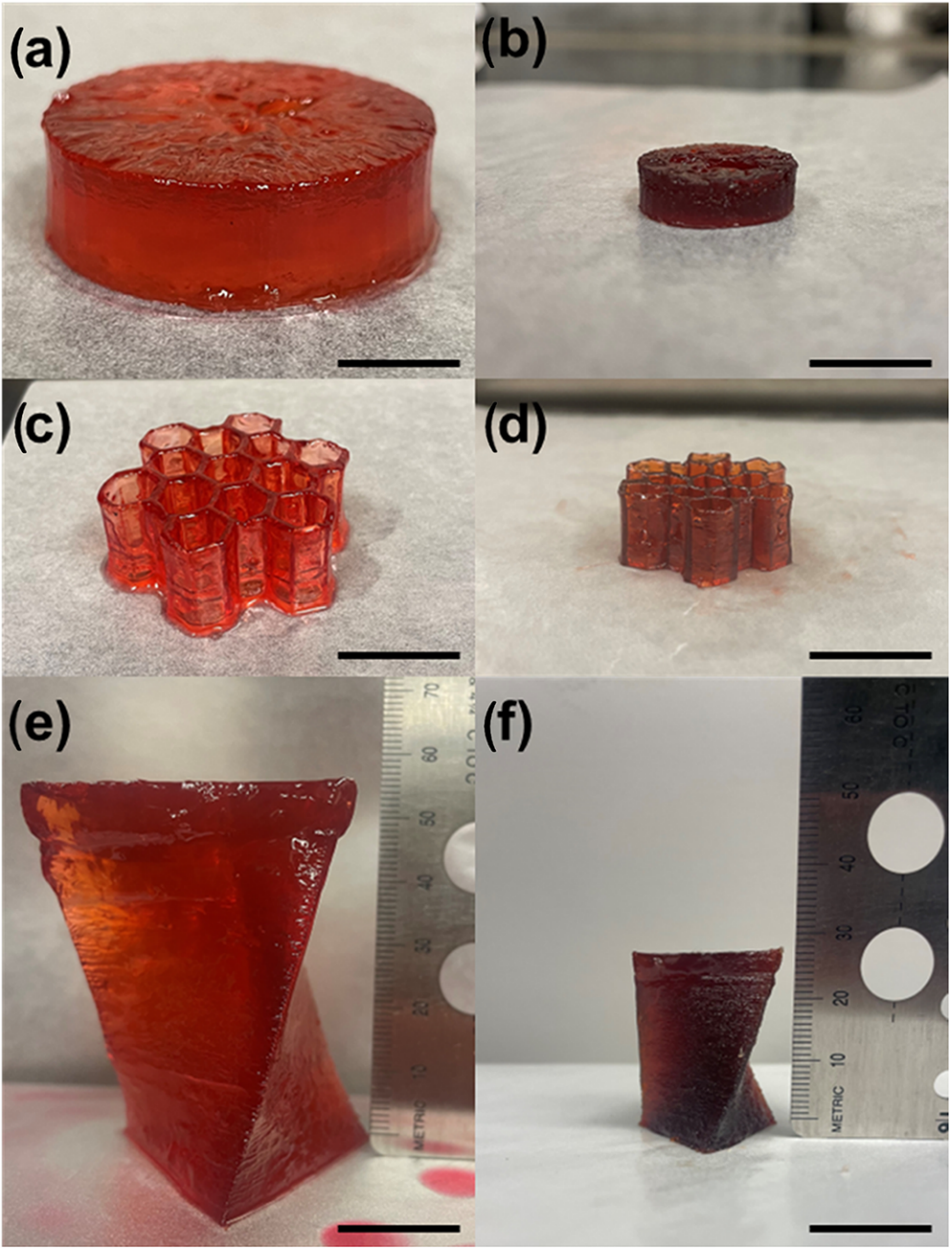
Printed geometries using 90 SH ODT formulation: (a) 2 cm tall cylinder as printed, (b) cylinder after solvent evaporation, (c) 2 cm tall hexagonal lattice as printed, (d) hexagonal lattice after solvent evaporation, (e) 6 cm tall, twisted prism as printed, and (f) twisted prism after solvent evaporation. Scale bars are all 2 cm.

## 4 Conclusions

A strategy for UV-curing polychloroprene was developed using thiol-ene chemistry to cure polychloroprene from solution and employed to additively manufacture articles using vat photopolymerization. Various thiols with differing functionalities were utilized as curatives, and UV-cured formulations with equimolar amounts of thiol functionality were used to investigate structure–property relationships. Thermal properties were largely unchanged across all formulations compared to the polychloroprene starting material. However, lower cross-linker concentrations enhanced ultimate tensile stress, strain at break, and toughness. This improvement is attributed to strain-induced crystallization, which is promoted by the increased molecular weight between cross-links observed at lower thiol concentrations. Photorheology experiments showed that cross-linker concentration had no effect on cure rate or structural rigidity of the printed networks. However, increasing the number of functional groups on the thiol cross-linker increased both cure rate and structural rigidity. All formulations were able to be printed using a DLP 3D printer, demonstrating that polychloroprene with varied properties can be successfully 3D printed in various shapes. Shrinkage of the articles after printing was quantified, and several strategies for reducing shrinkage were discussed. Overall, the additively manufactured polychloroprene elastomers, fabricated via vat photopolymerization, exhibited mechanical properties exceeding those of unfilled commercial polychloroprene rubber. The demonstrated mechanical performance, especially the ultrastretchable characteristics, which rival other high-performance elastomers and hydrogels, positions this thiol-ene polychloroprene system as a superior and versatile material for advanced elastomeric designs.

## Supplementary Material







## Notes

Detailed resin formulations, polymer solution viscosities, NMR spectra, and microstructure of polychloroprenes according to NMR data, FTIR spectra, properties and images of UV-cast samples, photorheology data, and properties and images of 3D-printed articles (PDF)
Video of 3D-printed dogbone being tested (MOV)
Time-lapse video of article shrinkage (MP4)
