# Supplementary material for: 3D-Printed, Ultrastretchable Polychloroprene Elastomers via Thiol-ene Photopolymerization
Source: ACS Appl Mater Interfaces. 2026 Apr 27;18(17):25278–86. doi: 10.1021/acsami.6c03614 (PMC13154126; doi:10.1021/acsami.6c03614)
Supplement: Supplementary file 1 [file am6c03614_si_001.pdf]

Supporting Information

3D-Printed, Ultra-Stretchable Polychloroprene Elastomers via Thiol-Ene Photopolymerization

Levi M. J. Moore[1‡], Maren E. Summers[2‡], Ashley M. Robinson[1], Anesia D. Auguste[3], Reagan Elia[1], Jared A. Gibson[4], Jacob C. Marcischak[1], Johnnay A. Martin[5], Kamran B. Ghiassi[1]*

[1] Aerospace Systems Directorate, Air Force Research Laboratory, Edwards AFB, CA 93524

[2] Amentum Holdings, Inc., Air Force Research Laboratory, Edwards AFB, CA 93524

[3] Materials and Manufacturing Directorate, Air Force Research Laboratory, Wright-Patterson AFB, Dayton, OH 45433

[4] AeroVironment, Inc., Air Force Research Laboratory, Wright-Patterson AFB, Dayton, OH 45433

[5] United States Air Force Academy, Colorado Springs, CO 80840

‡These authors contributed equally.

* ashley.robinson.29@us.af.mil

Table of Contents

1. Detailed Resin Formulations
2. Polymer Solution Viscosity
3. NMR Spectroscopy
4. FTIR Spectroscopy
5. UV-Cast Samples
6. Photorheology
7. 3D Printed Articles

1. Detailed Resin Formulations

Table S1. Detailed formulations for polychloroprene DLP resins. Formulations for UV-cast samples omit the Oil Red O.

Thiol	SH/chain	Mass Polymer Solution [g]	Mass Thiol [g]	Mass TPO [g]	Volume Oil Red O Solution [μL]
DT	30	40	0.194	0.069	-
	60	40	0.387	0.071	-
	90	40	0.581	0.072	-
ODT	30	40	0.101	0.068	265
	60	40	0.203	0.069	269
	90	40	0.304	0.070	273
TMPMP	30	40	0.148	0.068	267
	60	40	0.295	0.070	273
	90	40	0.443	0.071	279
PETMP	30	40	0.136	0.068	266
	60	40	0.271	0.069	272
	90	40	0.407	0.071	277

2. Polymer Solution Viscosity

Table S2. Polychloroprene:xylenes solution viscosity.

Mass ratio xylenes:PC	Viscosity [cP]
2:1	36690
3:1	3032
4:1	1163
5:1	174

3. NMR Spectroscopy

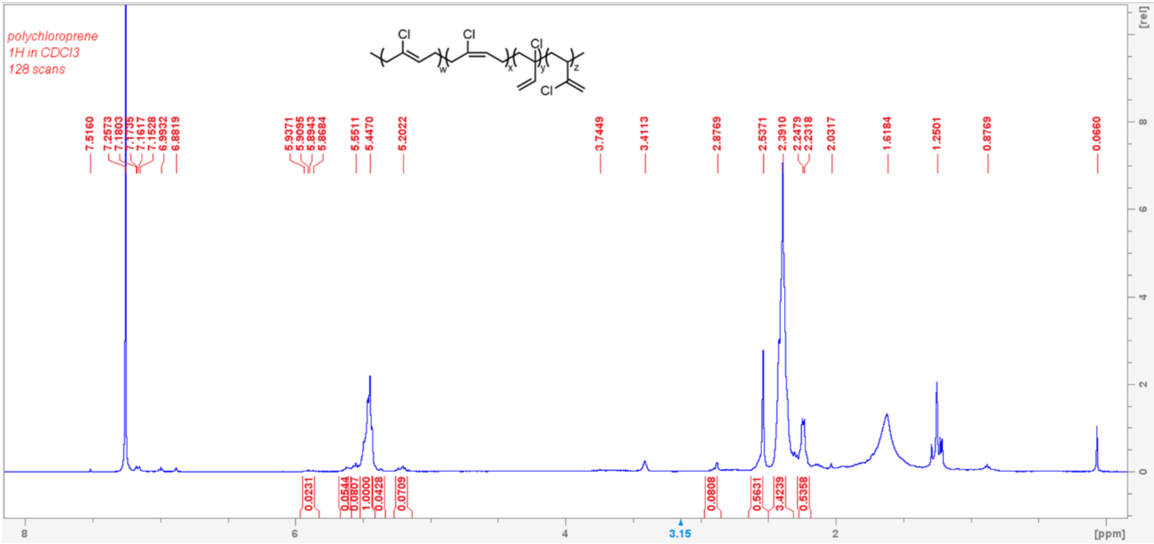

Figure S1. Polychloroprene, ^{1}H NMR spectrum in $CDCl_3$ 400MHz

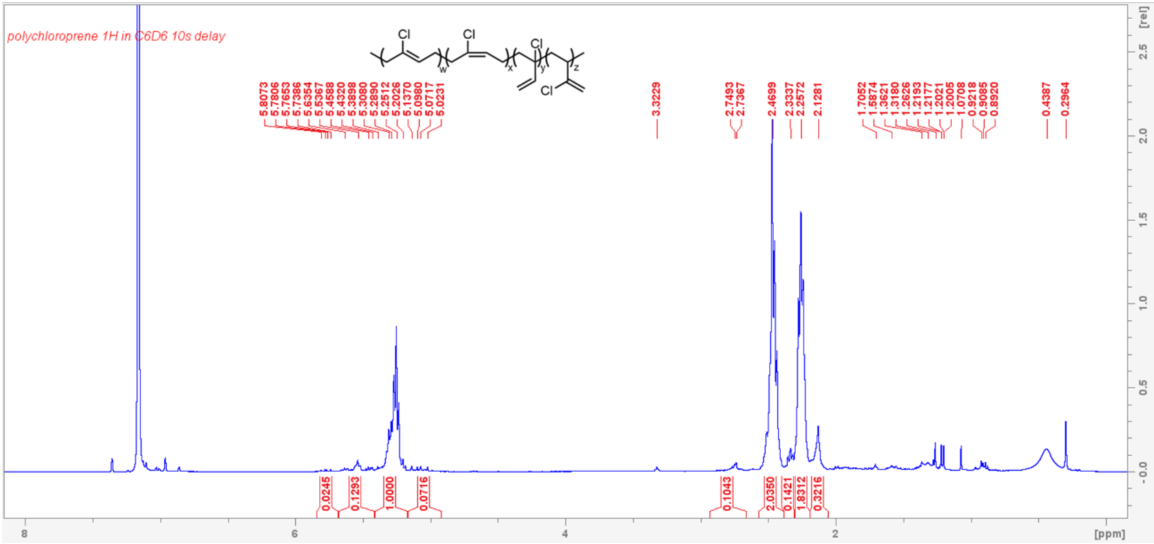

Figure S2. Polychloroprene, ^{1}H NMR spectrum in C_6D_6 400MHz

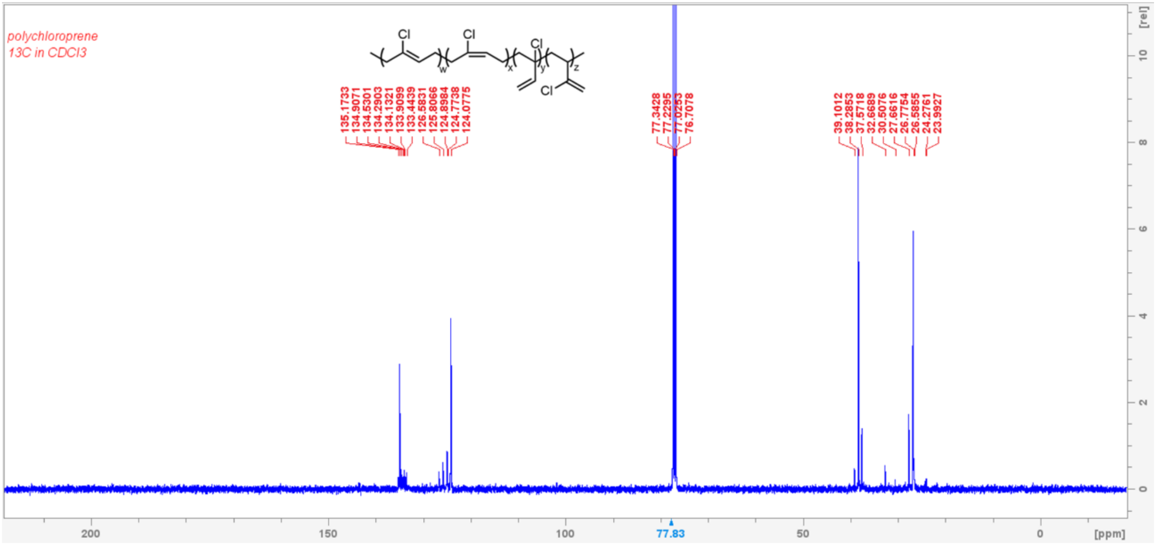

Figure S3. Polychloroprene, ^{13}C NMR spectrum in $CDCl_3$ 100MHz

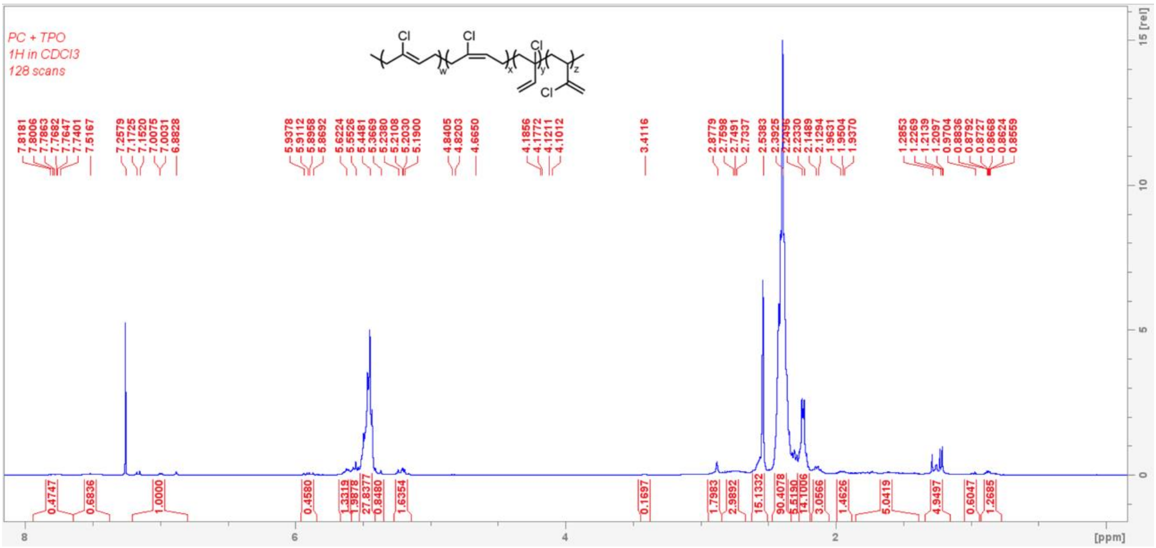

Figure S4. Polychloroprene + photoinitiator, ^{1}H NMR spectrum in $CDCl_3$ 400MHz

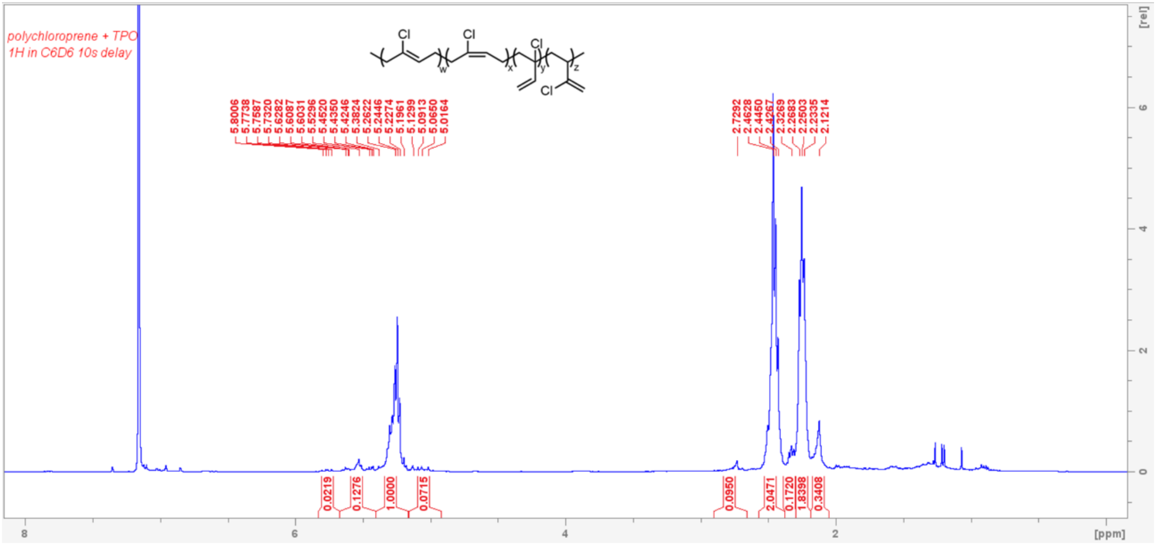

Figure S5. Polychloroprene + photoinitiator, ^{1}H NMR spectrum in C_6D_6 400MHz

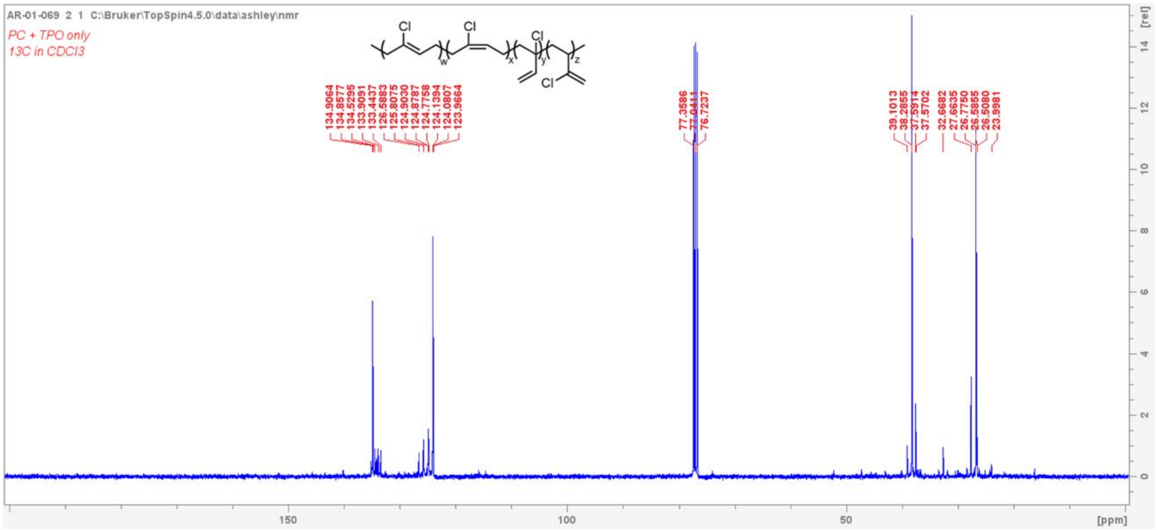

Figure S6. Polychloroprene + photoinitiator, ^{13}C NMR spectrum in $CDCl_3$ 100MHz

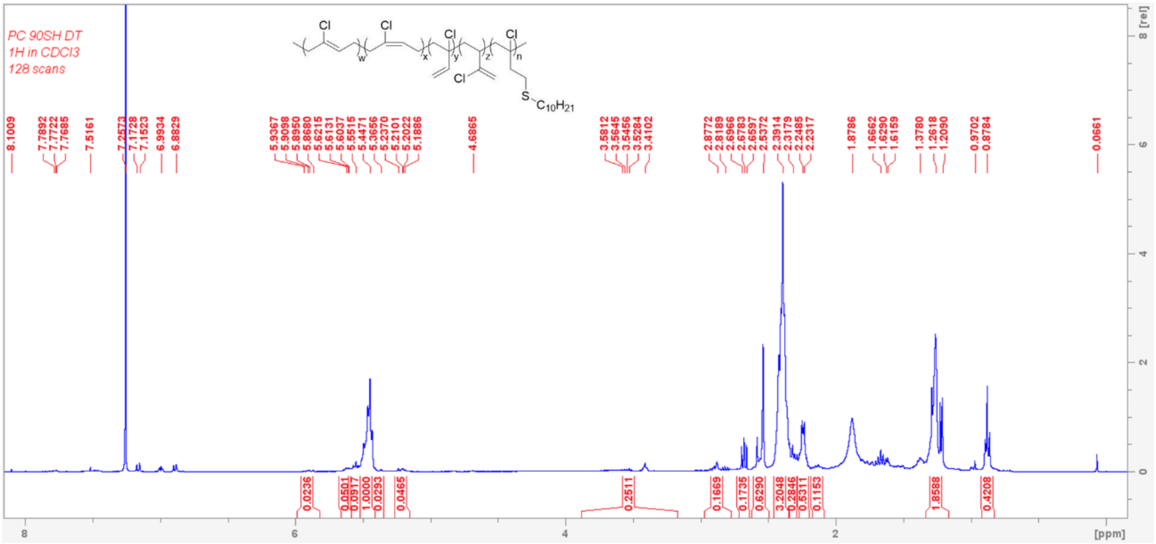

Figure S7. Polychloroprene + 90 SH decanethiol, soluble fraction, ^{1}H NMR spectrum in $CDCl_3$ 400MHz

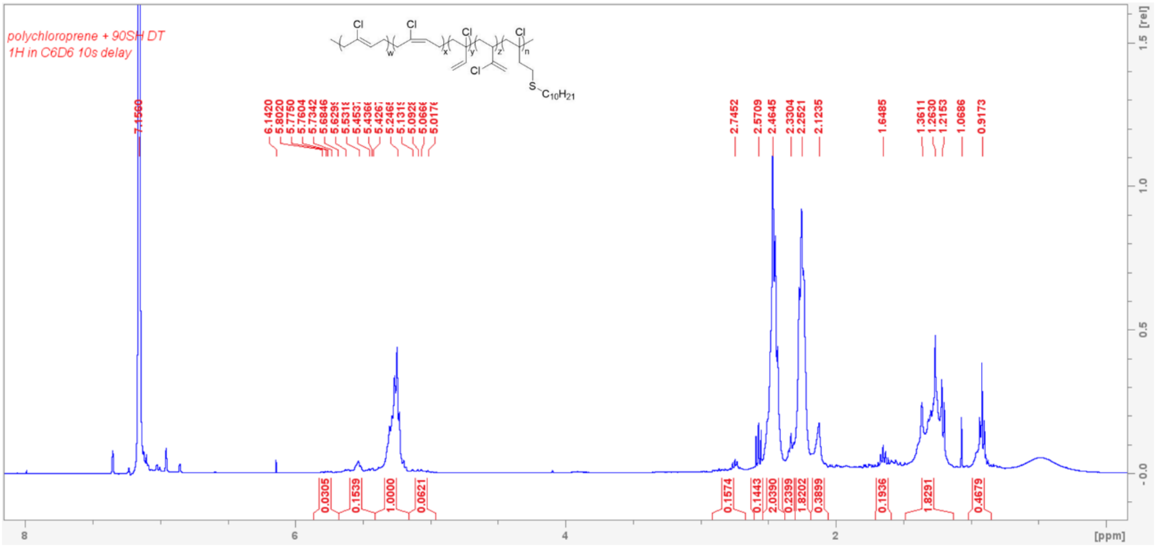

Figure S8. Polychloroprene + 90 SH decanethiol, soluble fraction, ^{1}H NMR spectrum in C_6D_6 400MHz

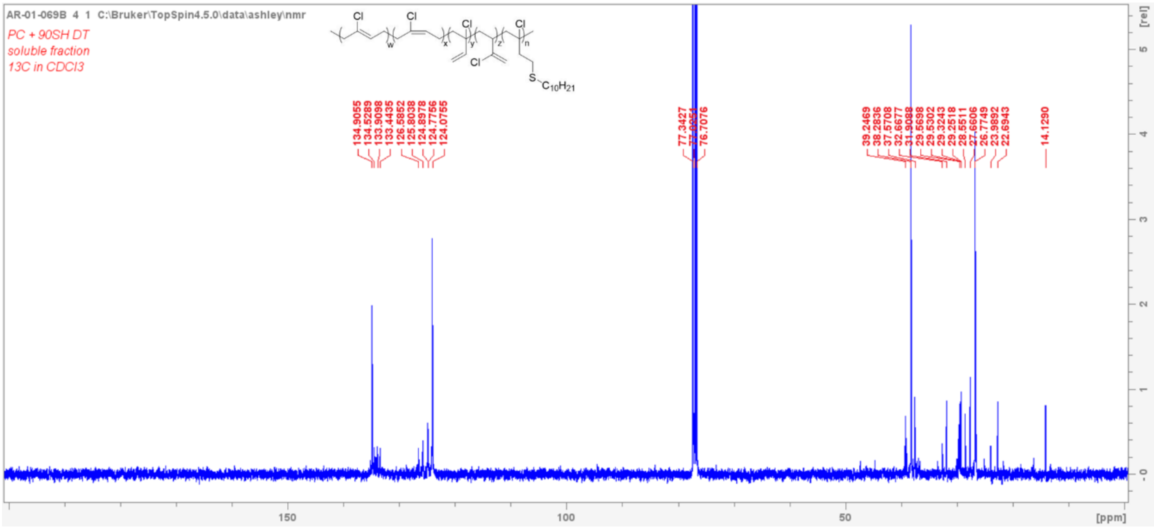

Figure S9. Polychloroprene + 90 SH decanethiol, soluble fraction, ^{13}C NMR spectrum in $CDCl_3$ 100MHz

Table S3. Microstructure of polychloroprenes according to ^{1}H NMR data

Sample	Ratio of aliphatic: olefinic protons	*cis*-content [mol %]	*trans*-content [mol %]	*vinyl*-content [mol %]
PC	3.62	11.2	84.6	4.2
PC + TPO	3.68	11.1	84.9	4.1
PC + TPO + DT	3.84	13.2	82.9	3.8

4. FTIR Spectroscopy

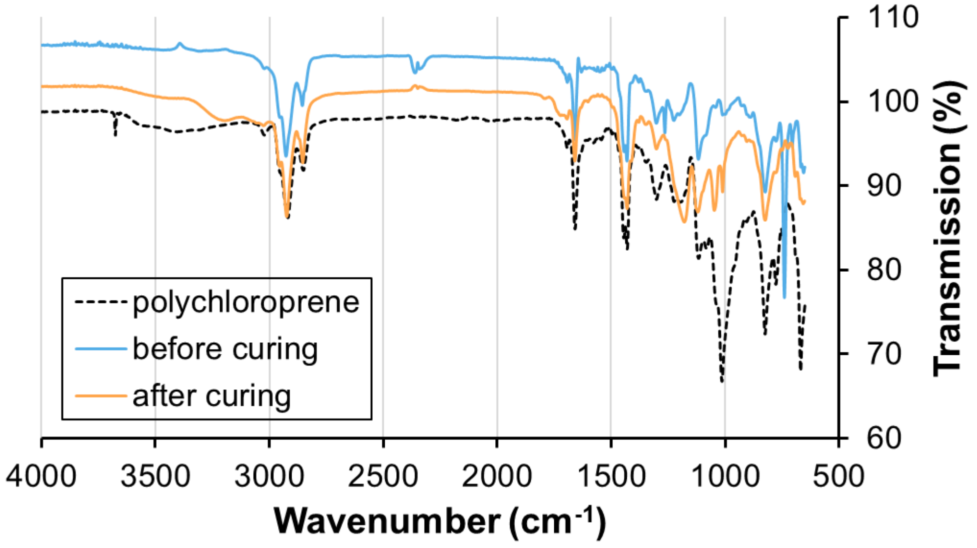

Figure S10. FTIR spectra of 90SH DT formulation before and after cure. Extra signals in "before curing" samples at 750 cm^{-1} and 1260 cm^{-1} are from residual dichloromethane used for solvent casting.

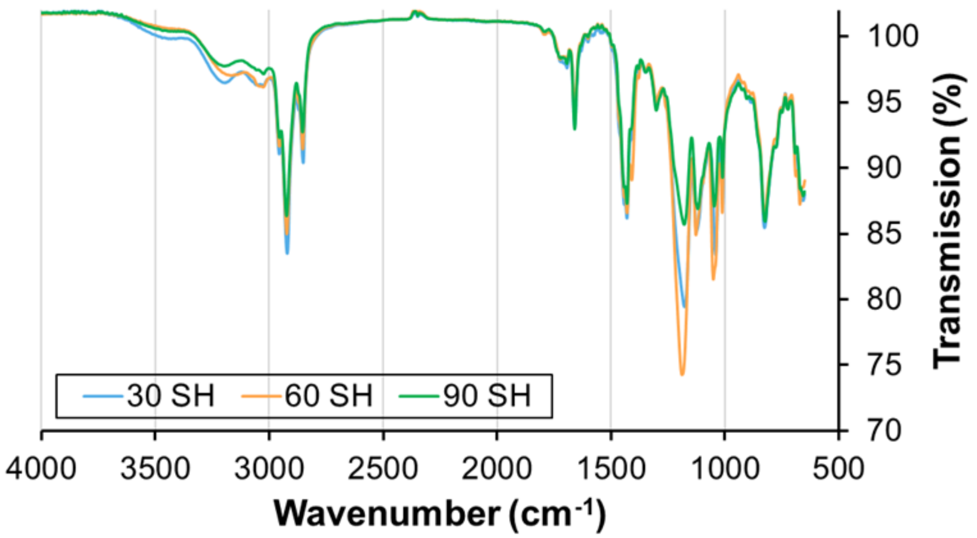

Figure S11. FTIR spectra of cured DT formulations.

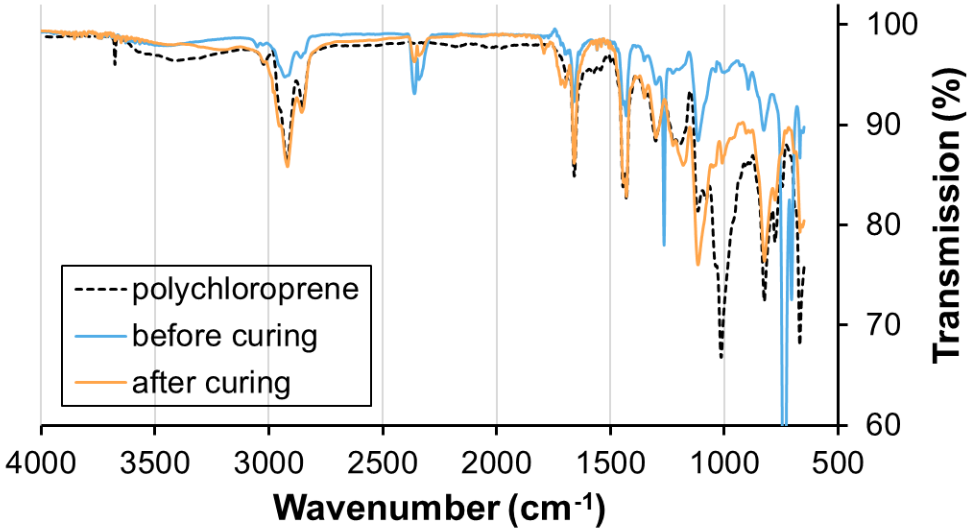

Figure S12. FTIR spectra of 90SH ODT formulation before and after cure. Extra signals in "before curing" samples at 750 cm^{-1} and 1260 cm^{-1} are from residual dichloromethane used for solvent casting.

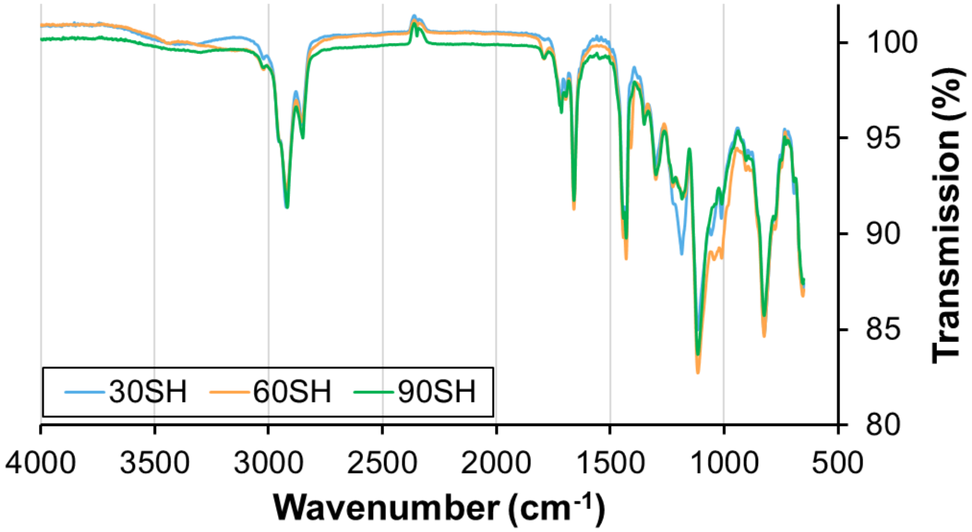

Figure S13. FTIR spectra of cured ODT formulations.

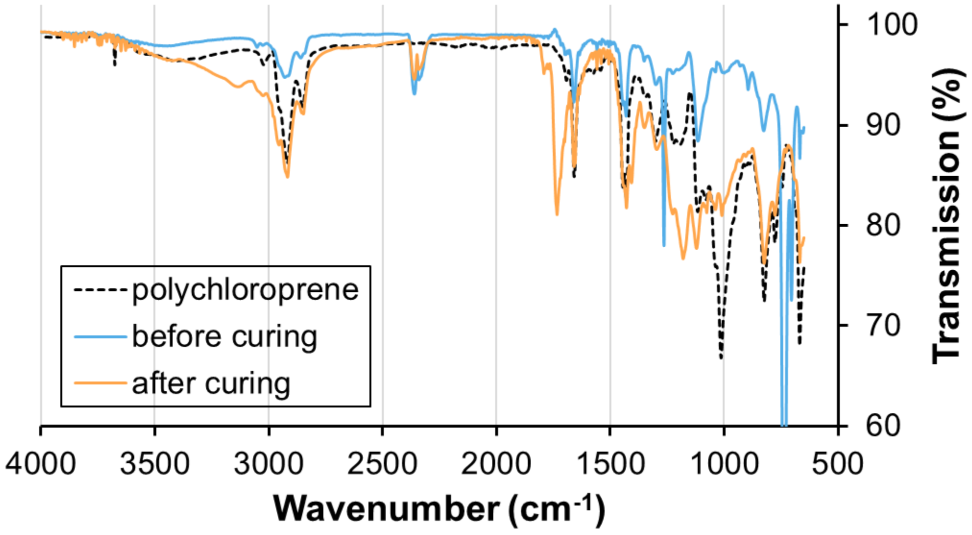

Figure S14. FTIR spectra of 90SH TMPMP formulation before and after cure. Extra signals in "before curing" samples at 750 cm^{-1} and 1260 cm^{-1} are from residual dichloromethane used for solvent casting.

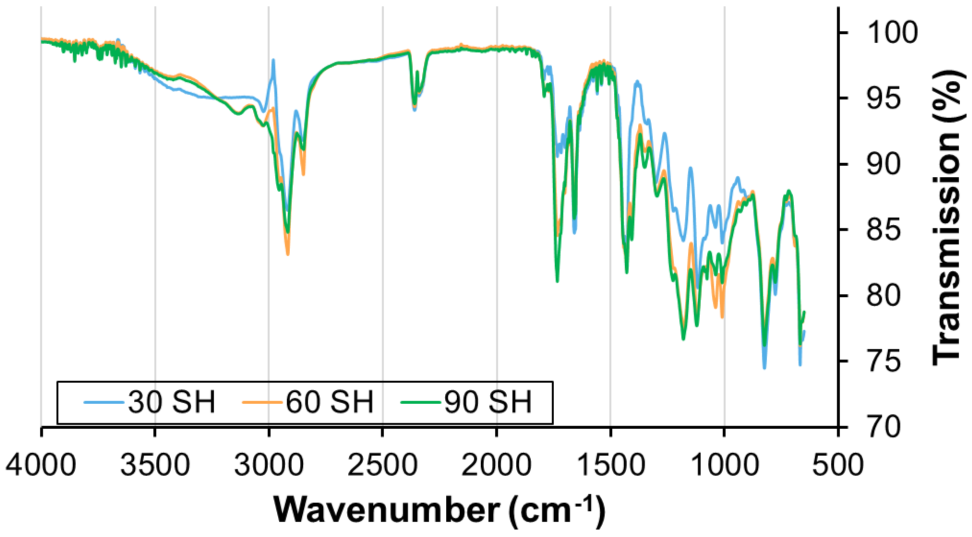

Figure S15. FTIR spectra of cured TMPMP formulations.

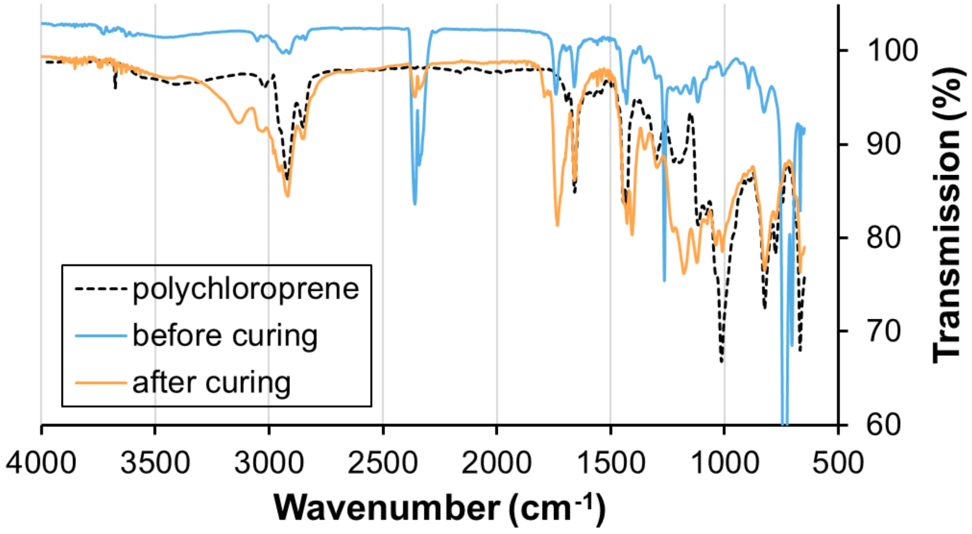

Figure S16. FTIR spectra of 90SH PETMP formulation before and after cure. Extra signals in "before curing" samples at 750 cm^{-1} and 1260 cm^{-1} are from residual dichloromethane used for solvent casting.

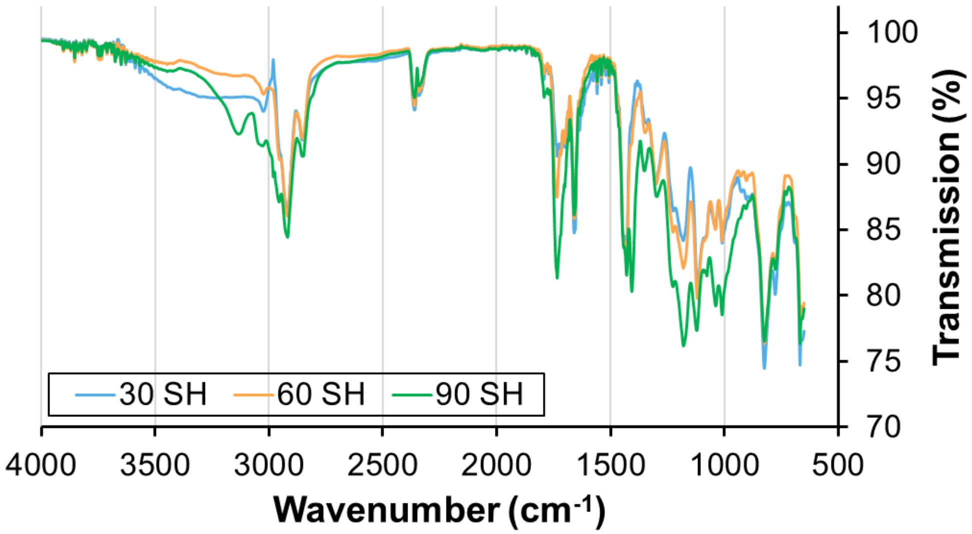

Figure S17. FTIR spectra of cured PETMP formulations.

5. UV Cast Samples

Table S4. Properties of UV-crosslinked polychloroprene with no thiol or decanethiol at several thiol loadings.

Thiol	Thiol loading [SH/chain]	Gel fraction [%]	T_g [°C]	$T_{d\,(5\%)}$, N_2 [°C]
Starting PC	--	--	-38.7 ±0.3	267
none	--	0 ±0	-36.8 ±0.1	258
DT	30	80.8 ±4.0	-37.4 ±1.0	245
	60	85.9 ±0.7	-40.3 ±0.2	226
	90	80.7 ±0.4	-41.6 ±0.3	227

Tensile Testing of Cast Samples

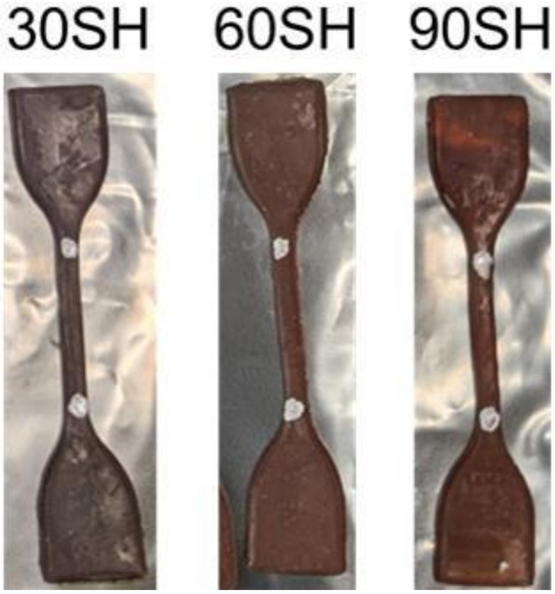

Figure S18. Cast tensile samples for formulations containing decanethiol in the ASTM D412 Type C geometry. As the solvent evaporated off the dogbones shrunk to a smaller version of that ASTM geometry.

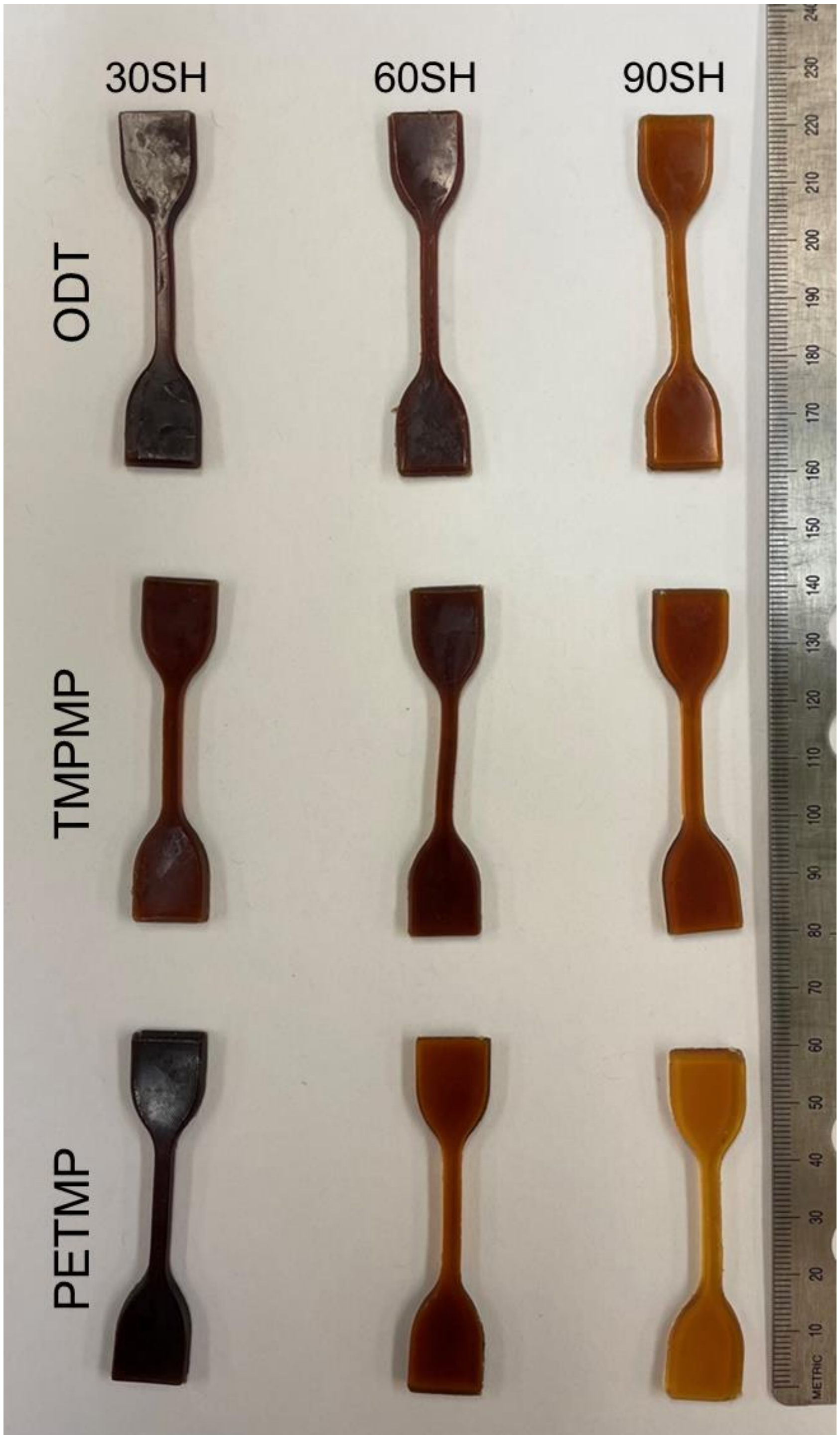

Figure S19. Cast tensile samples for all nine formulations containing ODT, TMPMP, or PETMP in the ASTM D412 Type C geometry. As the solvent evaporated off the dogbones shrunk to a smaller version of that ASTM geometry. Visually, higher loadings of thiol in the dogbones correspond to a lighter color in the cured article.

Table S5. Mechanical properties of UV-cast polychloroprene with different thiols and thiol loadings. Plus or minus values represent one standard deviation from a minimum of four samples.

Thiol	Thiol loading [SH/chain]	Swelling Ratio [%]	Shore A	Modulus [MPa]	Ultimate tensile stress [MPa]	Strain at break [%]	Toughness [MPa]	Yield Stress [MPa]
DT	30	1500 ±40	19.4 ±2.4	0.54 ±0.05	6.08 ±0.8	2010 ±96	33.2 ±5.0	0.11 ±0.01
	60	1360 ±30	17.7 ±2.2	0.47 ±0.03	3.62 ±0.5	2040 ±80	20.7 ±2.9	0.09 ±0.003
	90	2240 ±120	15.9 ±1.5	0.42 ±0.02	3.59 ±0.6	2150 ±140	21.2 ±3.9	0.09 ±0.003
ODT	30	1130 ±60	22.0 ±1.1	0.62 ±0.03	8.63 ±0.7	1820 ±76	44.6 ±6.1	0.15 ±0.006
	60	1170 ±15	21.1 ±1.2	0.61 ±0.04	2.64 ±0.2	1127 ±71	12.5 ±1.4	0.17 ±0.007
	90	1090 ±40	22.1 ±1.3	0.51 ±0.04	1.18 ±0.5	728 ±197	5.0 ±2.7	0.15 ±0.009
TMPMP	30	1150 ±50	21.9 ±1.0	0.71 ±0.06	4.13 ±0.9	1189 ±68	16.2 ±3.5	0.17 ±0.014
	60	860 ±50	22.2 ±0.5	0.73 ±0.05	1.11 ±0.1	475 ±17	3.1 ±0.3	0.19 ±0.009
	90	830 ±10	23.5 ±1.7	0.66 ±0.07	0.81 ±0.1	295 ±33	1.5 ±0.3	0.16 ±0.014
PETMP	30	1140 ±40	22.6 ±0.9	0.69 ±0.06	3.07 ±0.5	1109 ±70	13.3 ±2.3	0.17 ±0.01
	60	880 ±30	23.5 ±1.2	0.68 ±0.01	1.00 ±0.1	389 ±53	2.3 ±0.5	0.16 ±0.003
	90	690 ±20	26.5 ±1.5	0.75 ±0.05	0.99 ±0.1	278 ±13	1.6 ±0.1	0.20 ±0.014

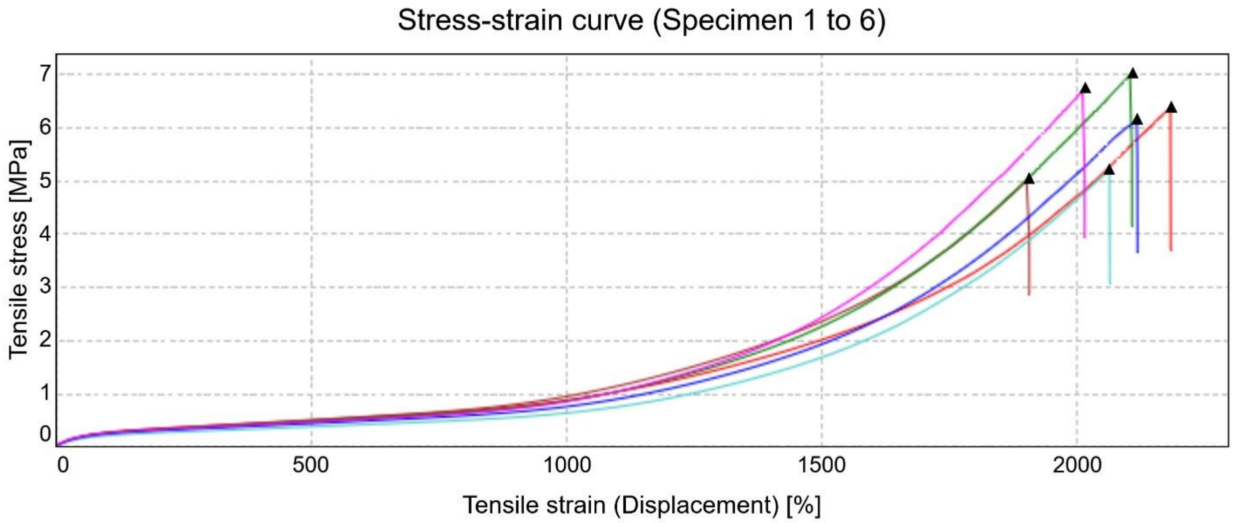

Figure S20. Stress-Strain plot for the crosslinked material of polychloroprene and DT at 30SH/polymer chain

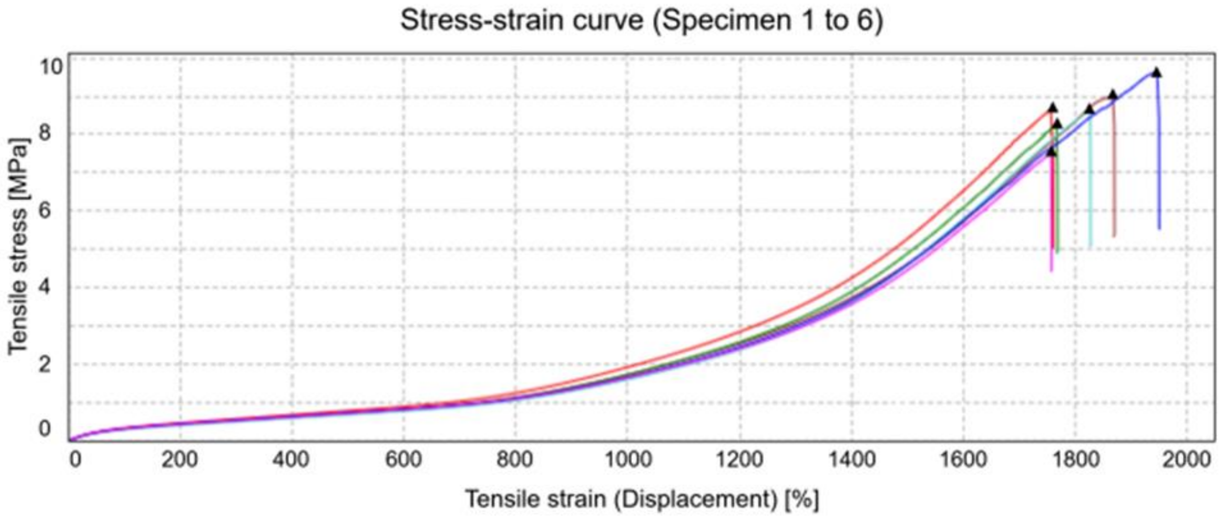

Figure S21. Stress-Strain plot for the crosslinked material of polychloroprene and ODT at 30SH/polymer chain

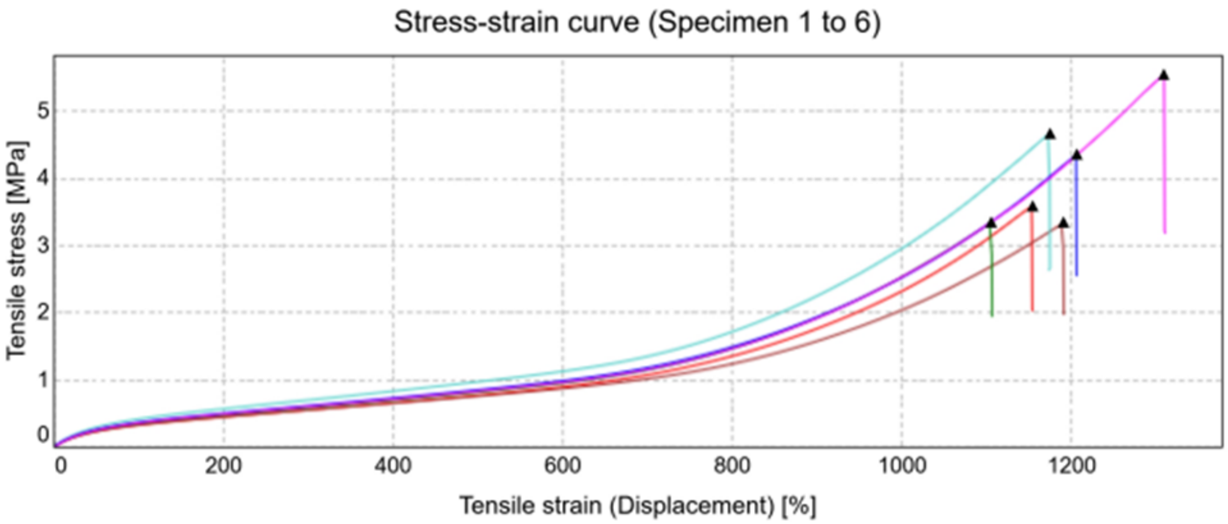

Figure S22. Stress-Strain plot for the crosslinked material of polychloroprene and TMPMP at 30SH/chain

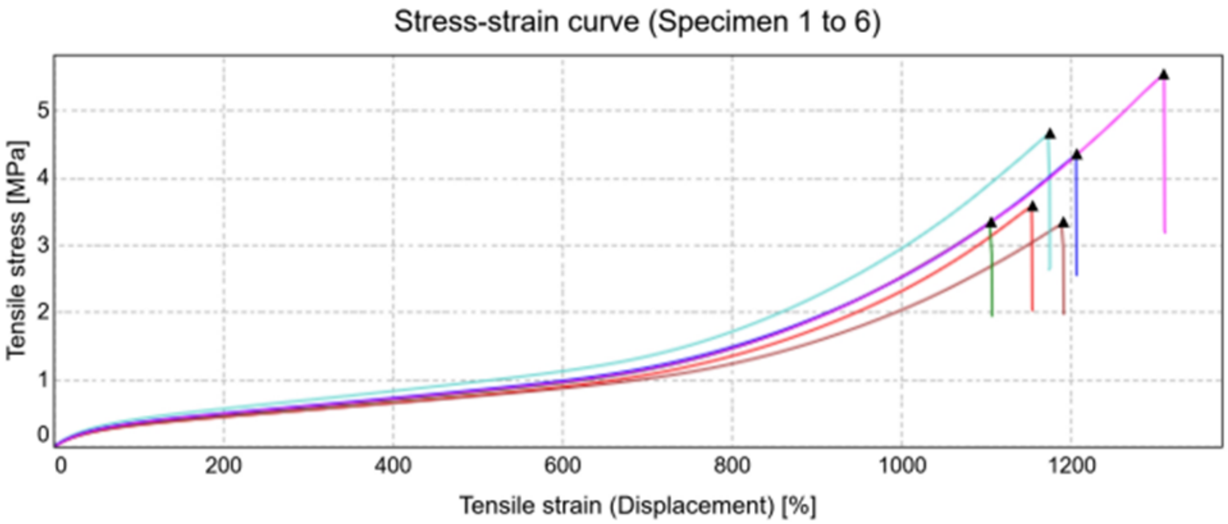

Figure S23. Stress-Strain plot for the crosslinked material of polychloroprene and PETMP at 30SH/chain

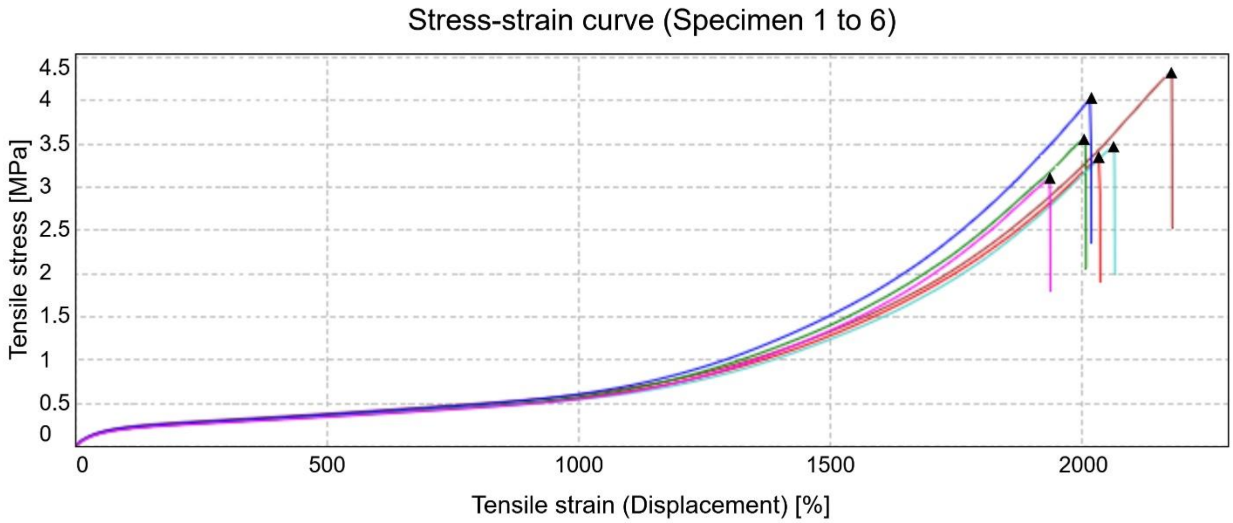

Figure S24. Stress-Strain plot for the crosslinked material of polychloroprene and DT at 60SH/polymer chain

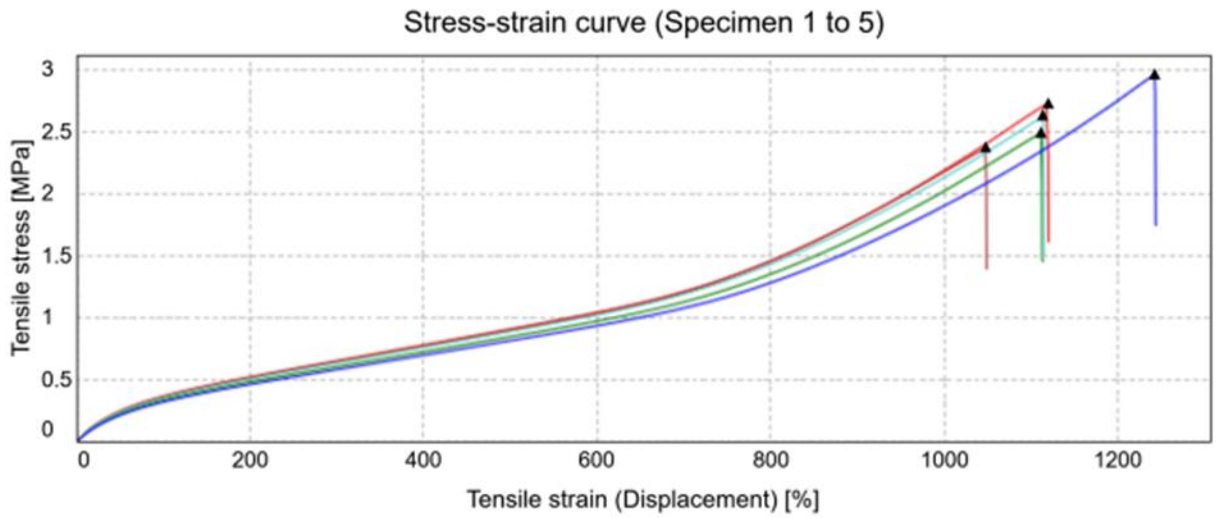

Figure S25. Stress-Strain plot for the crosslinked material of polychloroprene and ODT at 60SH/polymer chain

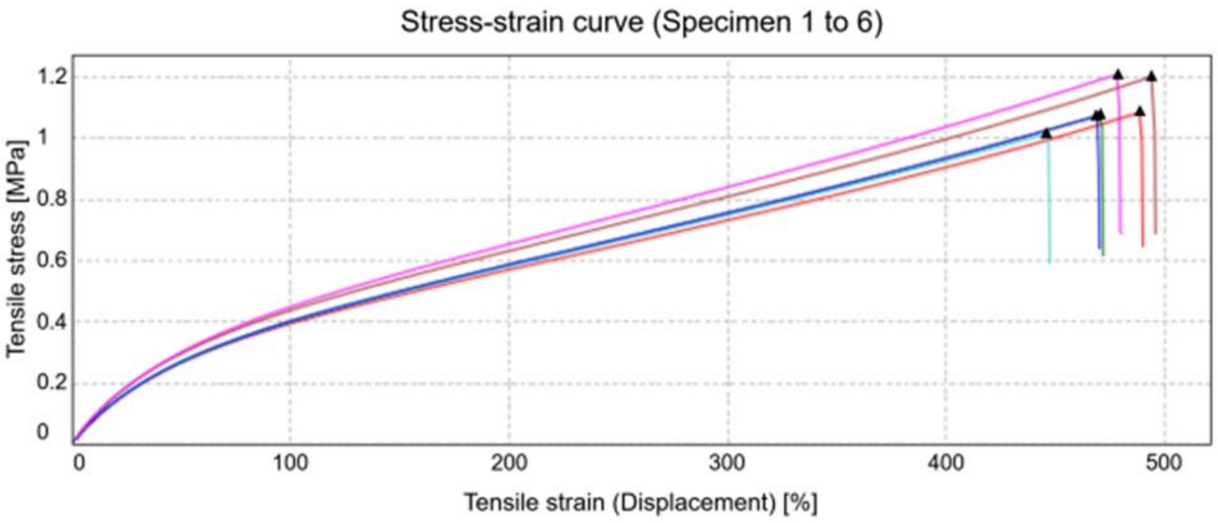

Figure S26. Stress-Strain plot for the crosslinked material of polychloroprene and TMPMP at 60SH/polymer chain

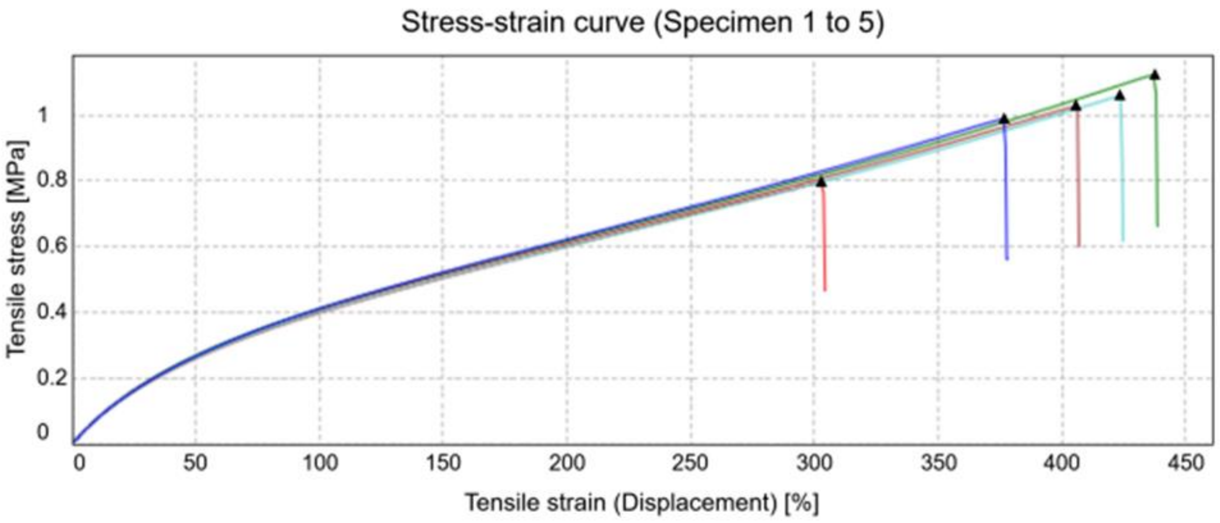

Figure S27. Stress-Strain plot for the crosslinked material of polychloroprene and PETMP at 60SH/chain

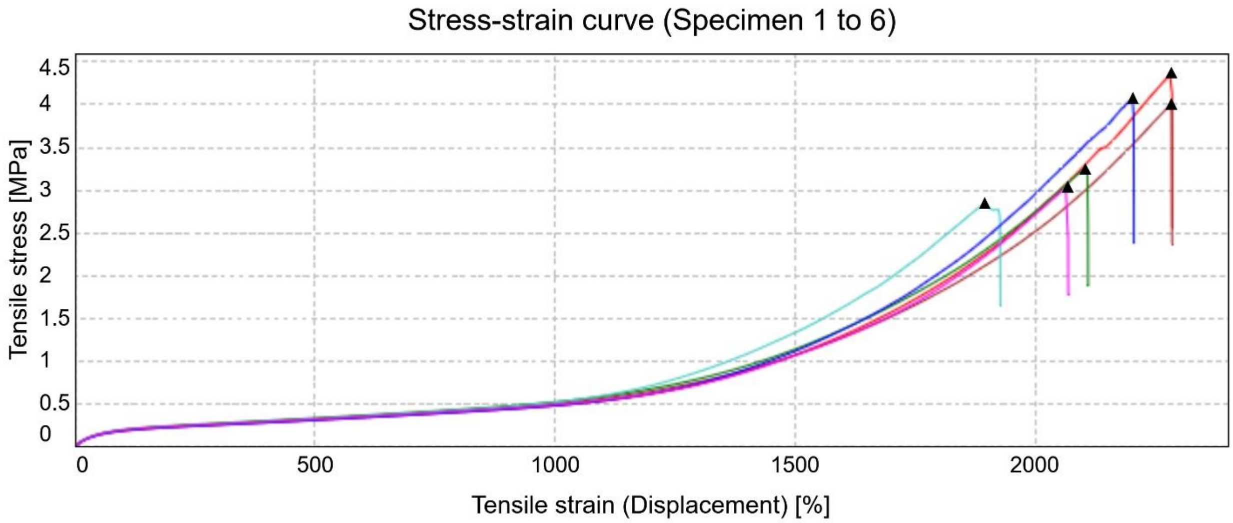

Figure S28. Stress-Strain plot for the crosslinked material of polychloroprene and DT at 90SH/polymer chain.

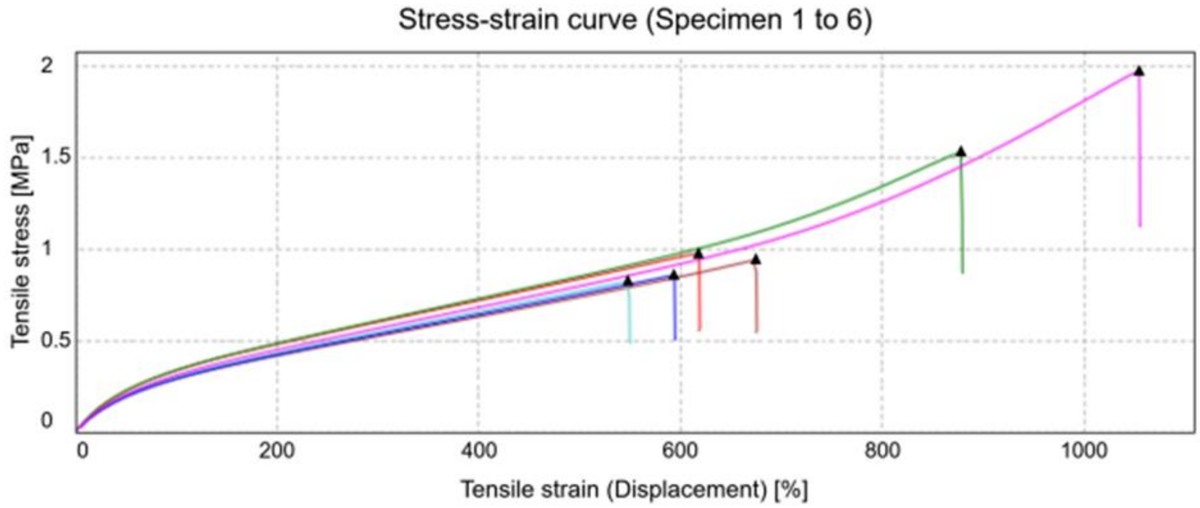

Figure S29. Stress-Strain plot for the crosslinked material of polychloroprene and ODT at 90SH/polymer chain.

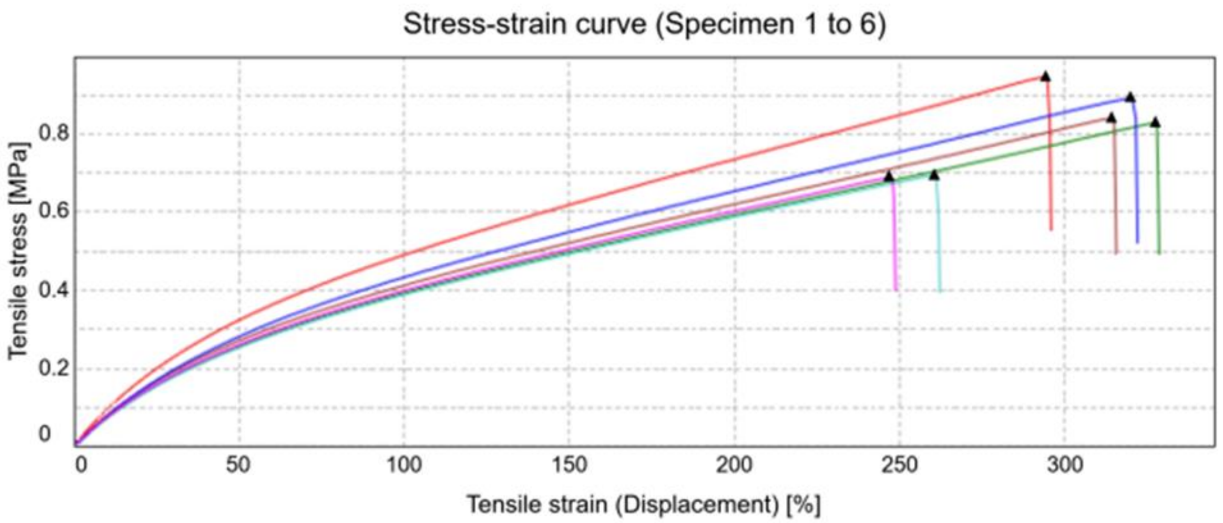

Figure S30. Stress-Strain plot for the crosslinked material of polychloroprene and TMPMP at 90SH/polymer chain.

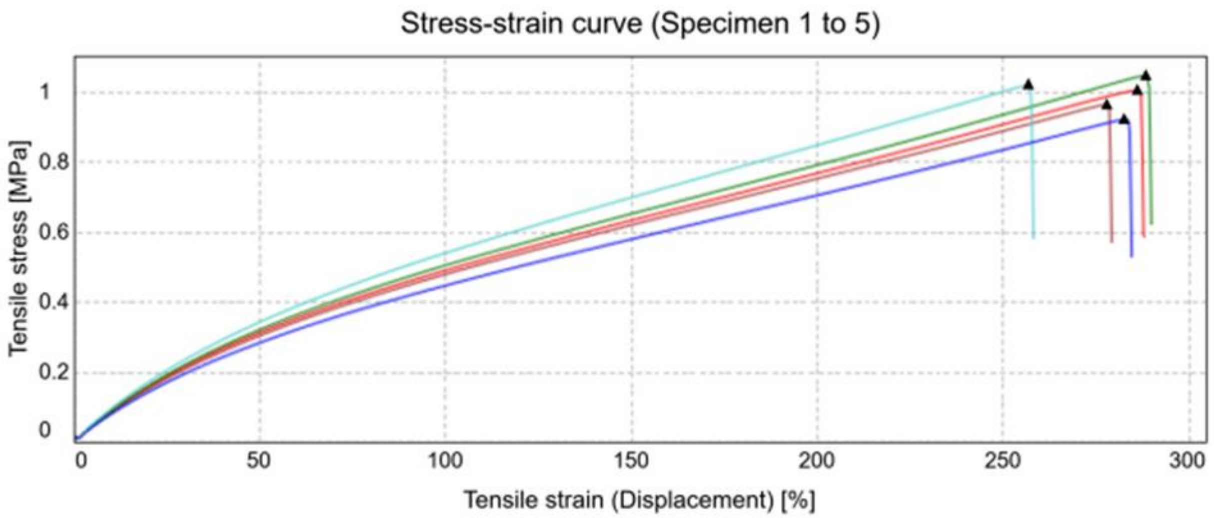

Figure S31. Stress-Strain plot for the crosslinked material of polychloroprene and PETMP at 90SH/polymer chain.

6. Photorheology

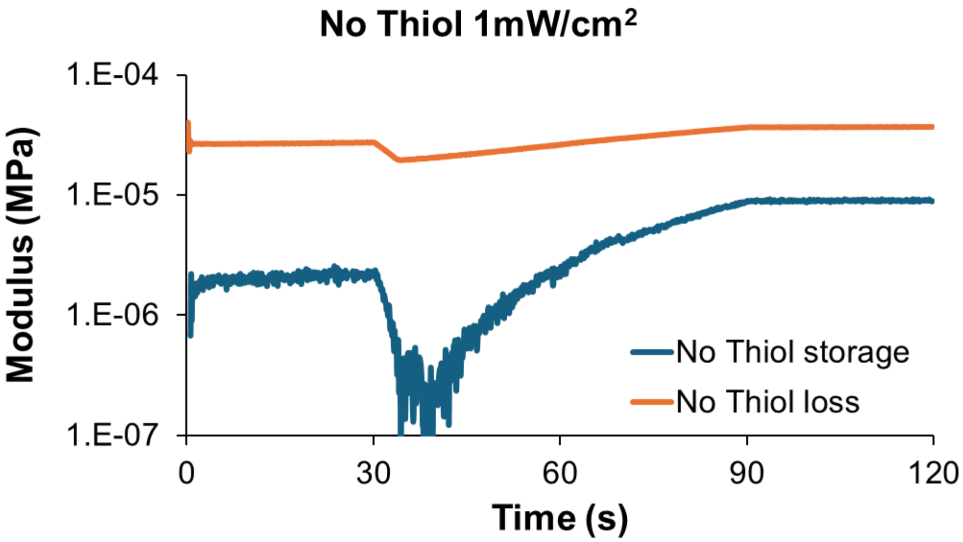

Figure S32. Photorheology of polychloroprene with TPO.

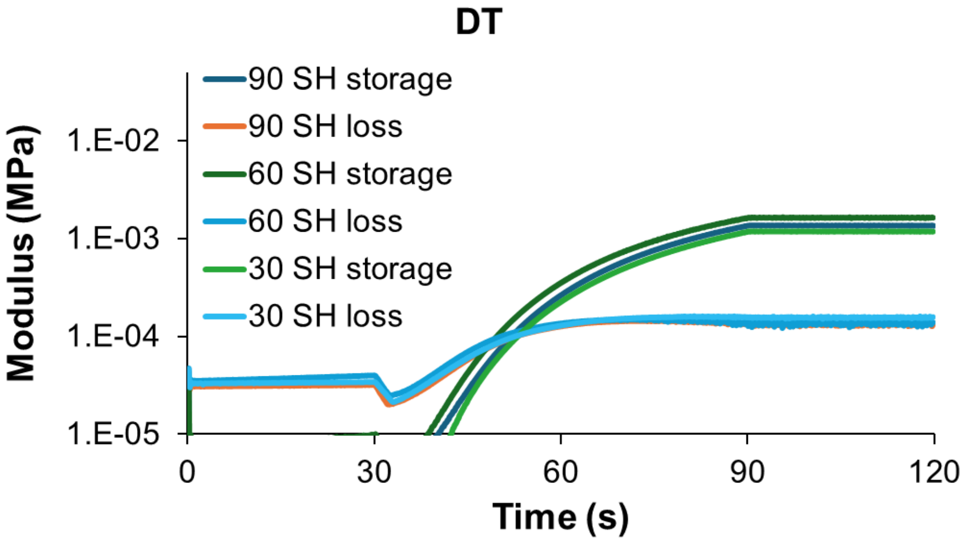

Figure S33. Photorheology of polychloroprene formulations containing DT.

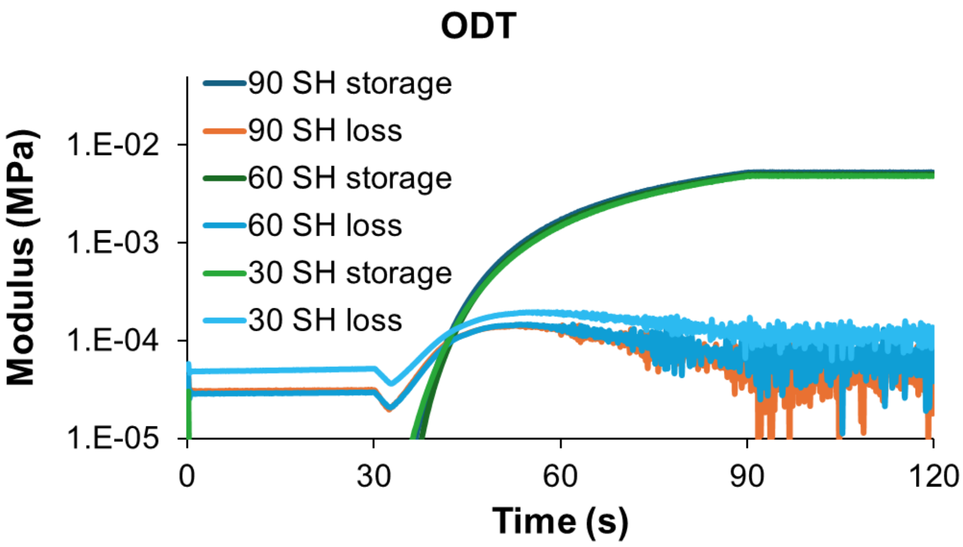

Figure S34. Photorheology of polychloroprene formulations containing ODT.

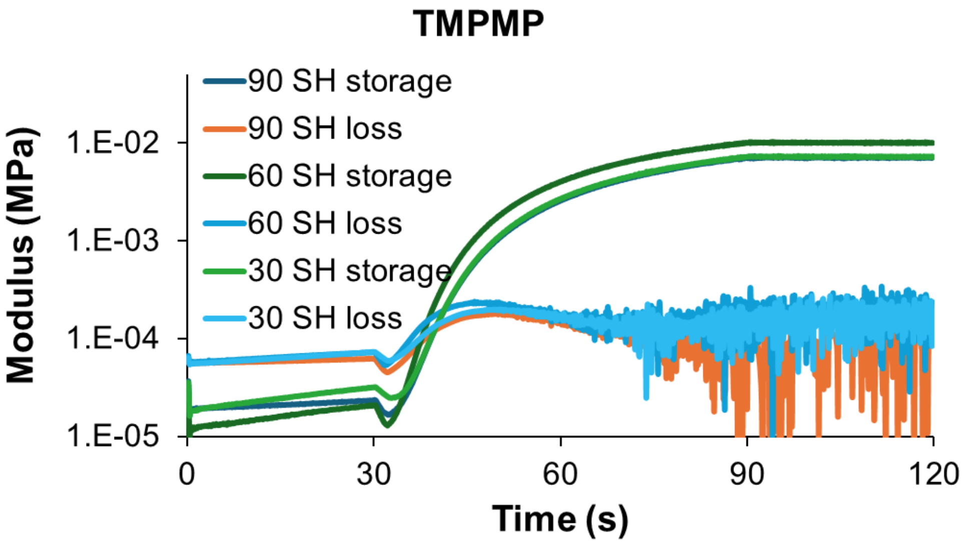

Figure S35. Photorheology of polychloroprene formulations containing TMPMP.

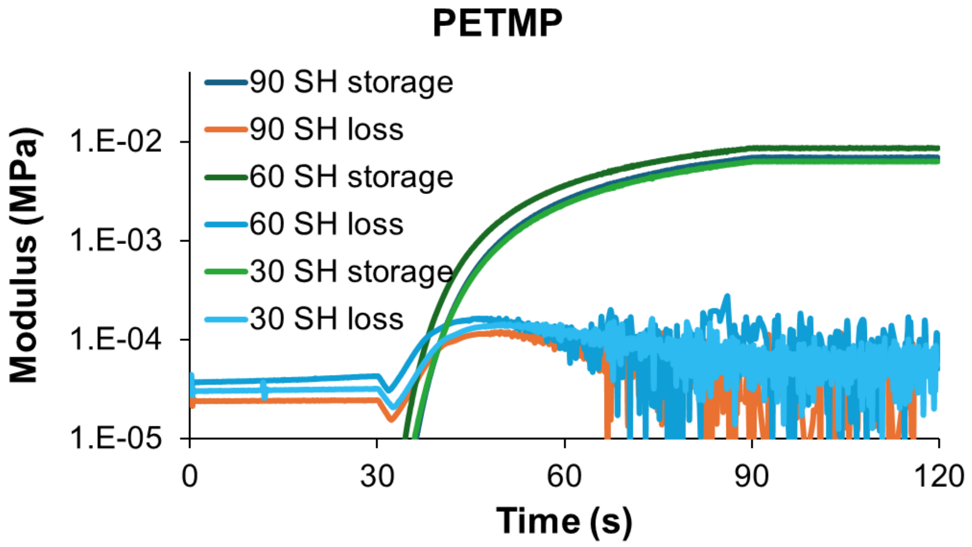

Figure S36. Photorheology of polychloroprene formulations containing PETMP.

7. 3D Printed Articles

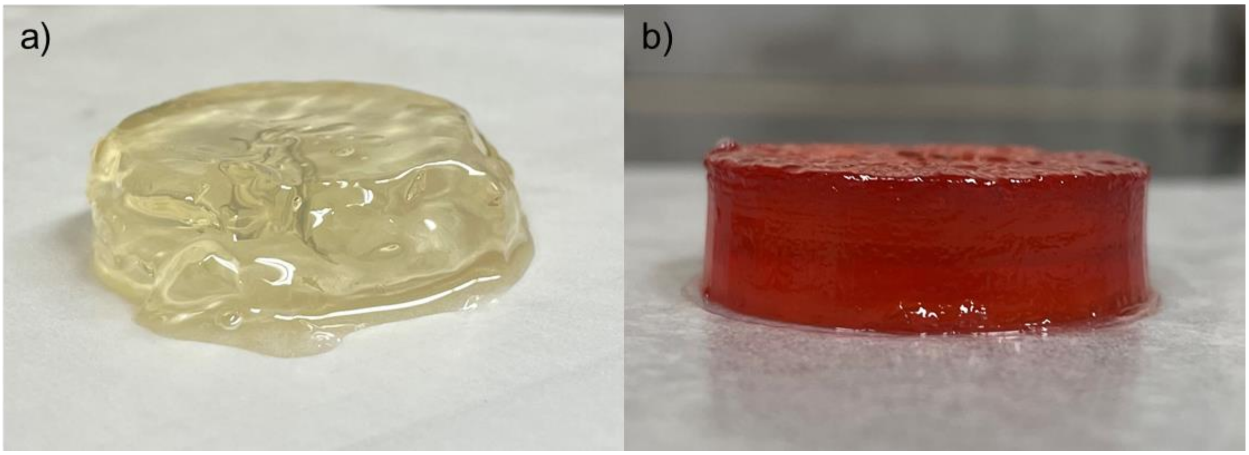

Figure S37. Before (a) and after (b) the addition of photoabsorber. The print in (a) has no photoabsorber and is comprised of the polychloroprene solution, ODT at 90SH/chain and TPO. The print in (b) is comprised of the aforementioned formulation with the addition of 0.04 wt% Oil Red O. Pictures were taken while prints were fresh off the build plate and solvent had not yet evaporated.

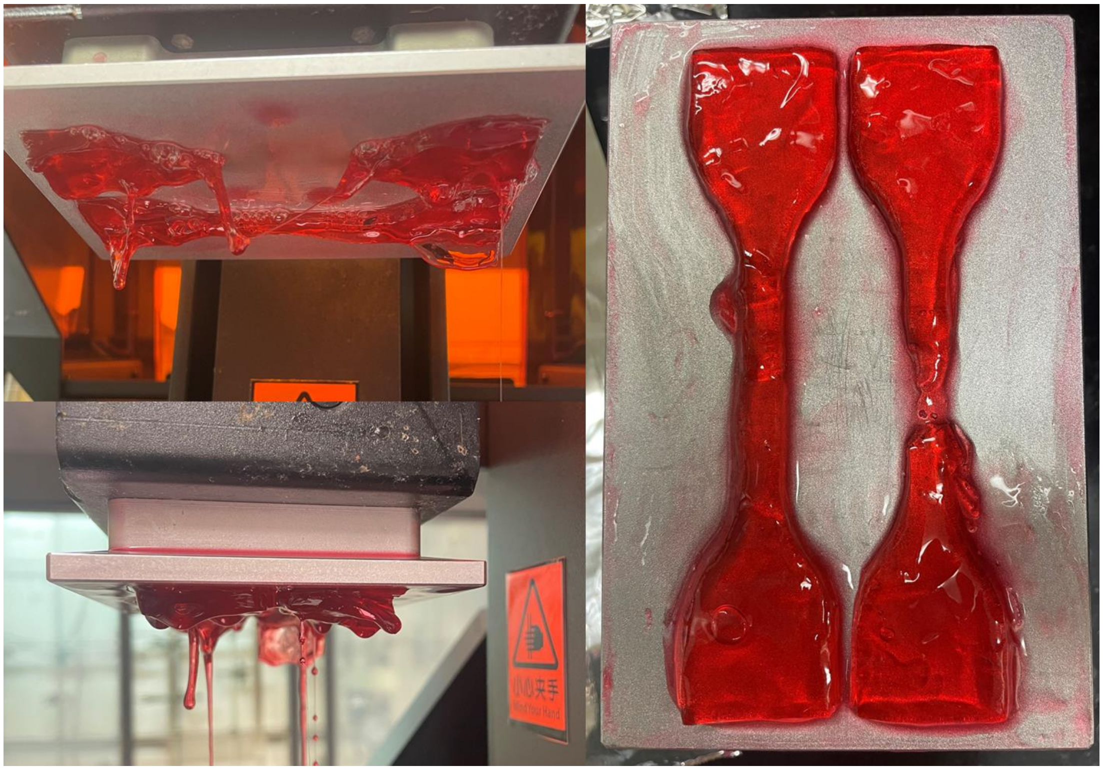

Figure S38. Printing with decanethiol was unsuccessful due to poor structural rigidity during printing.

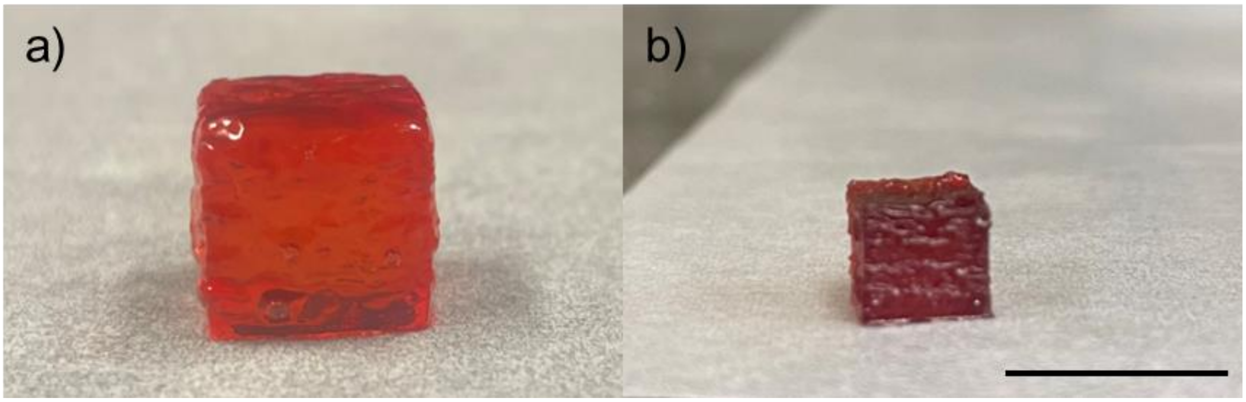

Figure S39. (a) Before and (b) after solvent evaporation of the 1 cm cube printed to quantify shrinkage. Reference bar in (b) is 1 cm.

Table S6. Quantitative data from the printed cube study.

Cube Type	Dimensions [mm]							Initial Volume [mm^3]	Final Volume [mm^3]	Volume Lost [%]
	Designed	As printed			After shrinking					
		x	y	z	x	y	z			
1 cm	10	9.98 ±0.06	9.94 ±0.08	9.33 ±0.44	5.45 ±0.25	5.36 ±0.21	4.85 ±0.08	926.5 ±51.5	141.8 ±13.0	84.7 ±1.1
2 cm	20	19.89 ± 0.08	19.63 ±0.25	18.50 ±0.42	10.55 ±0.07	10.51 ±0.11	10.12 ±0.20	7224.0 ±282.0	1122.4 ±40.4	84.5 ±0.1

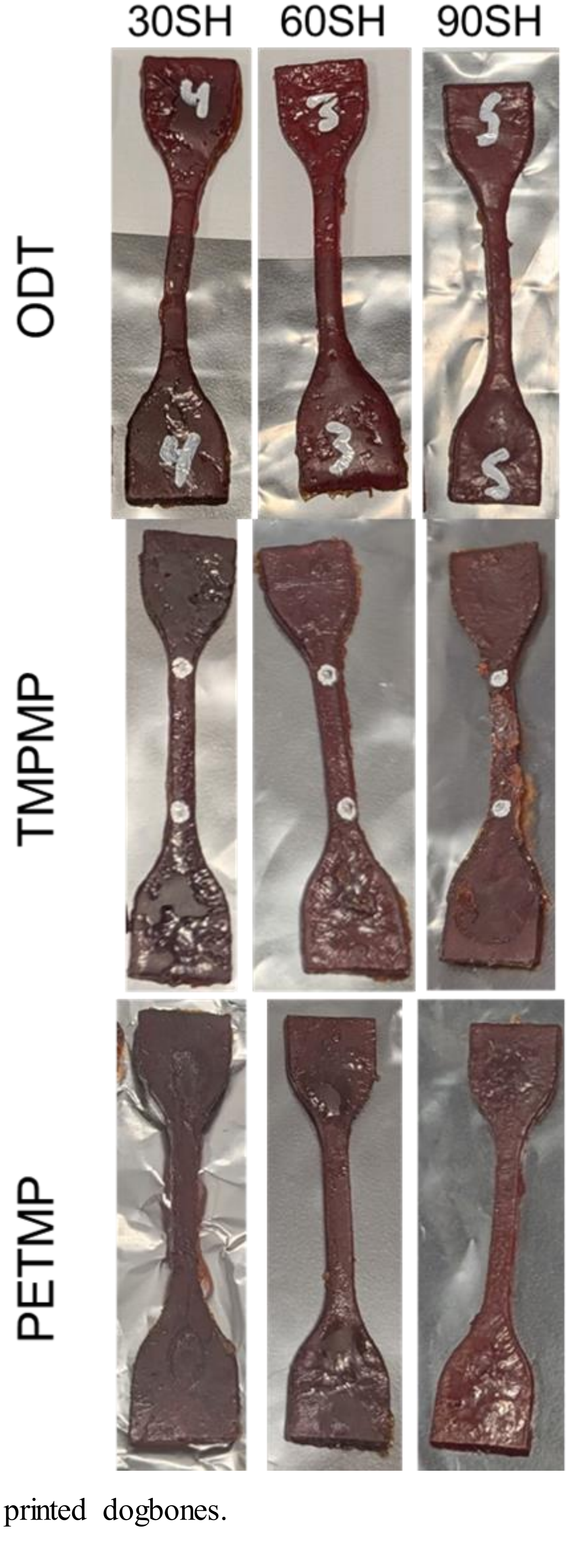

Figure S40. Picture of 3D printed dogbones.

Table S7. Properties of 3D printed UV-crosslinked polychloroprene with different thiols and thiol loadings. Plus or minus values represent one standard deviation from a minimum of four samples.

Thiol	Thiol loading [SH/chain]	Gel fraction [%]	Swelling Ratio [%]	Shore A	Young's Modulus [MPa]	Ultimate tensile stress [MPa]	Strain at break [%]	Toughness [MPa]	Yield Stress [MPa]
ODT	30	78.0 ±2.2	1580 ±140	12.4 ±2.6	1.13 ±0.12	2.42 ±1.25	1210 ± 325	13.9 ±7.9	0.17 ± 0.01
	60	84.9 ±0.7	1230 ±50	13.5 ±2.1	0.94 ±0.24	1.14 ±0.20	675 ±124	4.90 ±1.4	0.18 ± 0.03
	90	89.8 ±0.7	900 ±50	15.4 ±2.6	0.81 ±0.11	0.97 ±0.05	472 ±37.7	2.98 ±0.30	0.20 ± 0.01
TMPMP	30	81.4 ±1.0	1380 ±120	13.0 ±2.5	1.65 ±0.12	1.71 ±0.27	847 ±122	8.44 ±1.91	0.22 ± 0.002
	60	89.7 ±0.9	870 ±60	13.8 ±1.6	0.95 ±0.12	1.00 ±0.08	446 ±48.2	2.92 ±0.50	0.21 ± 0.02
	90	91.2 ±0.5	770 ±40	17.2 ±3.0	0.90 ±0.176	0.77 ±0.13	238 ±26.0	1.23 ±0.32	0.20 ± 0.02
PETMP	30	87.7 ±0.6	1130 ±30	13.8 ±2.1	1.29 ±0.17	1.12 ±0.20	506 ±137	3.87 ±1.52	0.23 ± 0.02
	60	89.5 ±1.2	750 ±80	15.0 ±1.3	1.07 ±0.10	0.88 ±0.06	293 ±51.3	1.70 ±0.37	0.20 ± 0.01
	90	90.1 ±0.8	700 ±10	15.3 ±3.0	1.02 ±0.13	0.91 ±0.09	225 ±42.5	1.30 ±0.32	0.22 ± 0.01

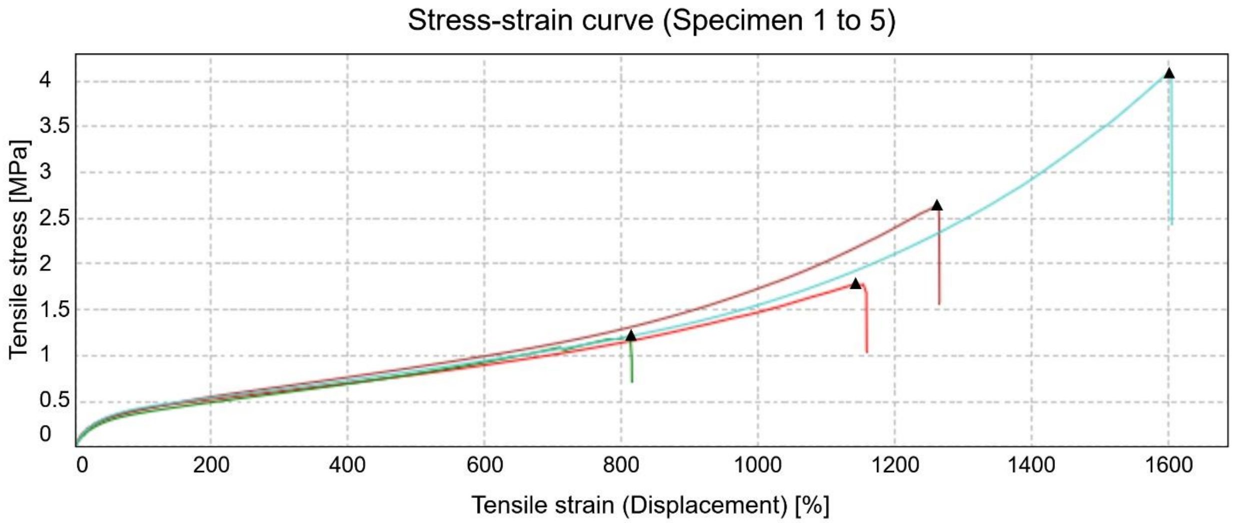

Figure S41. Stress-strain plot for the 3D printed UV-crosslinked material of polychloroprene and ODT at 30SH/polymer chain.

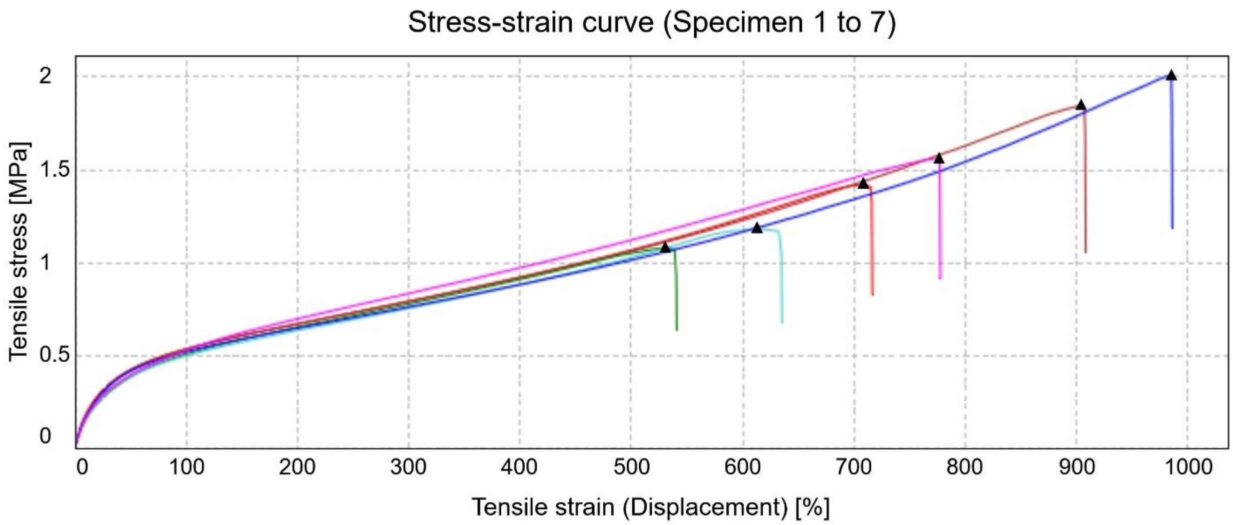

Figure S42. Stress-strain plot for the 3D printed UV-crosslinked material of polychloroprene and TMPMP at 30SH/polymer chain.

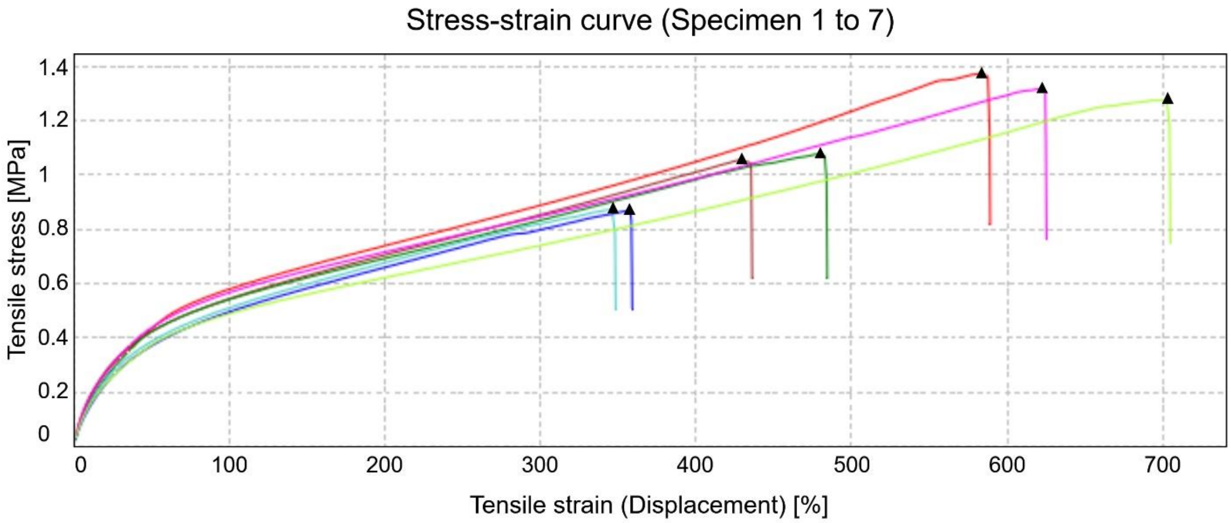

Figure S43. Stress-strain plot for the 3D printed UV-crosslinked material of polychloroprene and PETMP at 30SH/polymer chain.

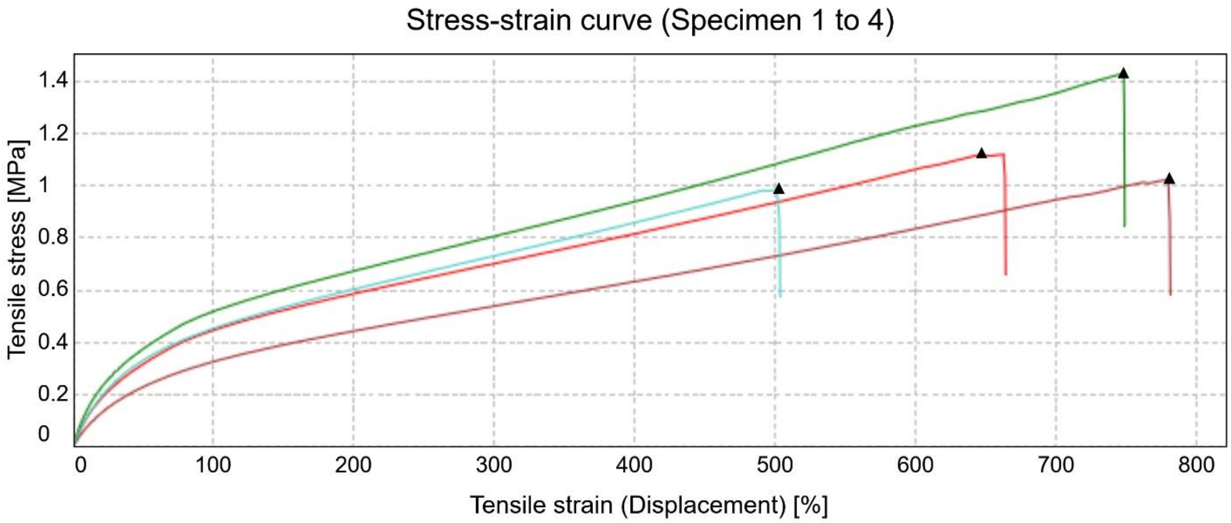

Figure S44. Stress-strain plot for the 3D printed UV-crosslinked material of polychloroprene and ODT at 60SH/polymer chain.

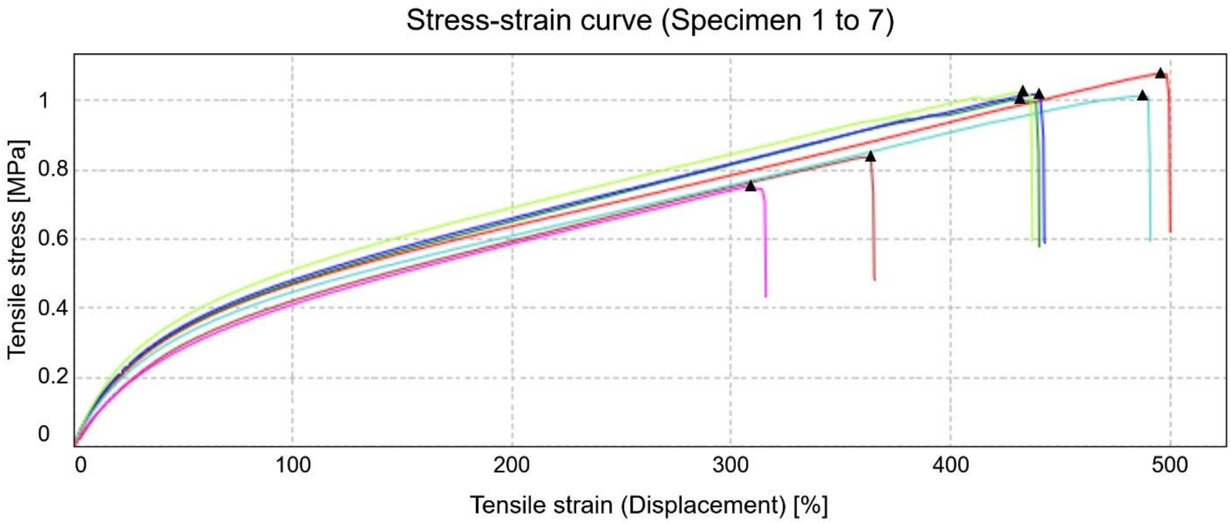

Figure S45. Stress-strain plot for the 3D printed UV-crosslinked material of polychloroprene and TMPMP at 60SH/polymer chain.

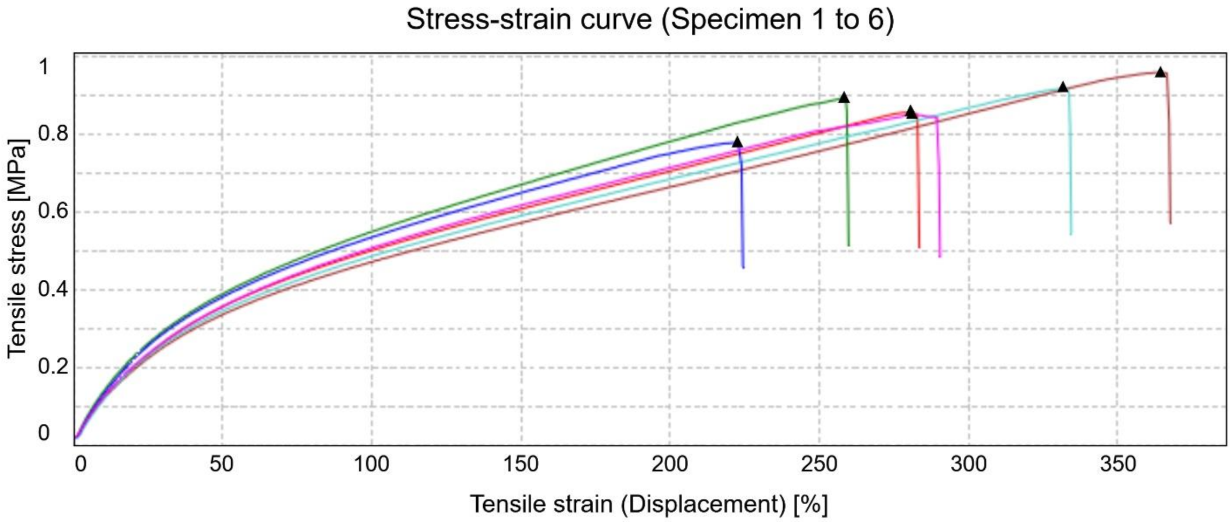

Figure S46. Stress-strain plot for the 3D printed UV-crosslinked material of polychloroprene and PETMP at 60SH/polymer chain.

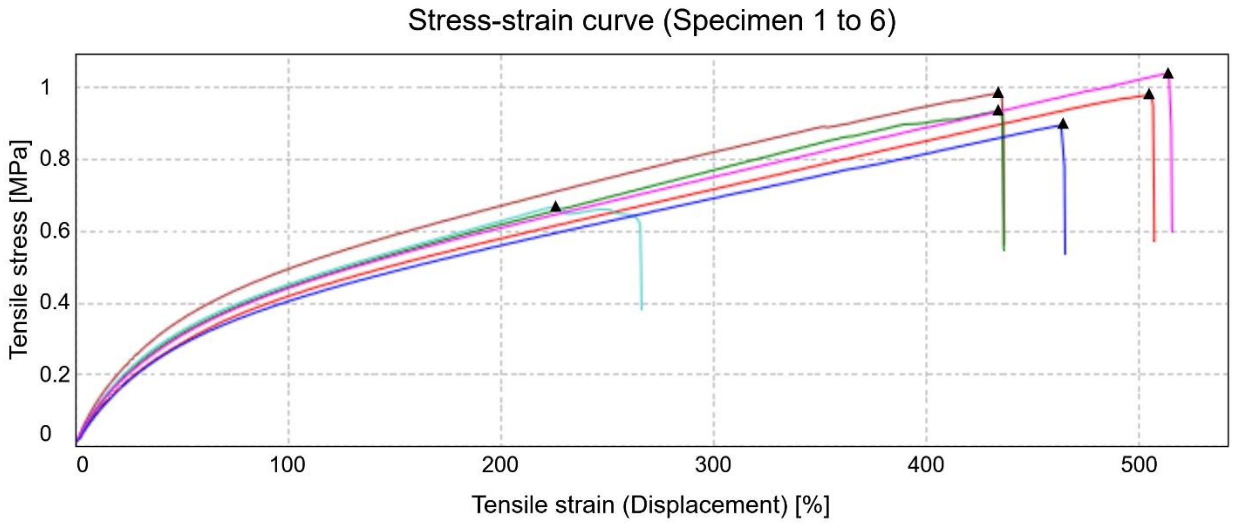

Figure S47. Stress-strain plot for the 3D printed UV-crosslinked material of polychloroprene and ODT at 90SH/polymer chain.

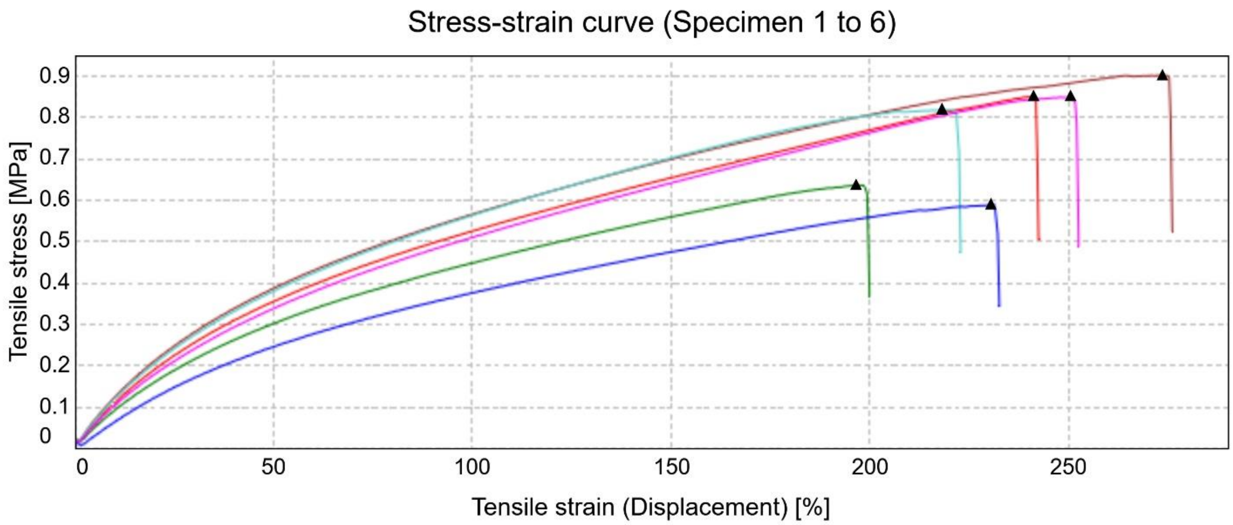

Figure S48. Stress-strain plot for the 3D printed UV-crosslinked material of polychloroprene and TMPMP at 90SH/polymer chain.

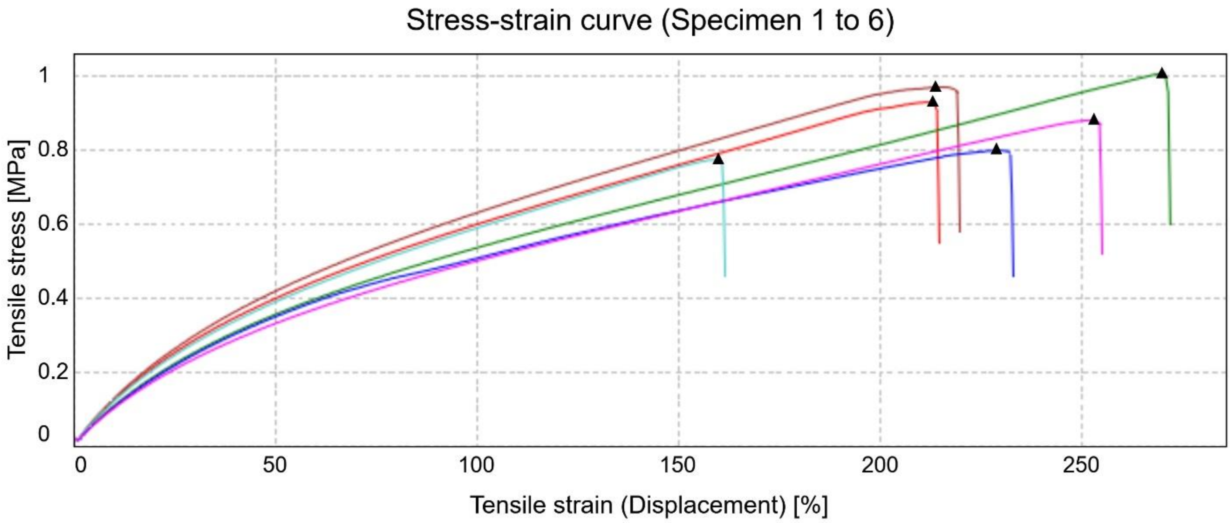

Figure S49. Stress-strain plot for the 3D printed UV-crosslinked material of polychloroprene and PETMP at 90SH/polymer chain.
